# RNA toehold switch-based reporter assay to assess bacterial uptake of antisense oligomers

**DOI:** 10.1128/mbio.03983-24

**Published:** 2025-03-04

**Authors:** Paramita Sarkar, Linda Popella, Sandra Pérez-Jiménez, Jörg Vogel

**Affiliations:** 1 Institute for Molecular Infection Biology (IMIB), Faculty of Medicine, University of Würzburg, Würzburg, Germany; 2 Cluster for Nucleic Acid Therapeutics Munich (CNATM), Munich, Germany; 3 Helmholtz Institute for RNA-based Infection Research (HIRI), Helmholtz Centre for Infection Research (HZI), Würzburg, Germany; Universite de Geneve, Geneva, Switzerland

**Keywords:** antisense oligomers, peptide nucleic acids, reporter assay, toehold switch, cell-penetrating peptides, alternative antibiotics

## Abstract

Antisense oligomers (ASOs) hold promise as antibiotics for the selective targeting of bacterial pathogens and as tools for the modulation of gene expression in microbes that are not amenable to genetic engineering. However, their efficient delivery across the complex bacterial envelope remains a major challenge. There are few methods to assess the efficiency of carrier-mediated ASO uptake by bacteria. Here, we have developed a “switch-on” reporter assay to measure ASO uptake efficiency in a semi-quantitative manner. The assay uses a synthetic RNA toehold switch fused to the mRNA of a fluorescent reporter protein, which is activated *in vivo* by a peptide nucleic acid (PNA)-based ASO upon delivery into the bacterial cytosol. We have used this assay to screen different cell-penetrating peptides (CPPs) as ASO carriers in *Escherichia coli* and *Salmonella enterica* and observed up to 60-fold activation, depending on the CPP and bacterial strain used. Our assay shows high dynamic range and sensitivity, which should enable high-throughput screens for bacterial ASO carriers. We also show that the reporter can be used to study routes of PNA uptake, as demonstrated by reduced reporter activity in the absence of the inner membrane protein SbmA. In summary, we present a tool for the discovery of species-specific and efficient ASO carriers that will also be useful for a broader investigation of cellular uptake mechanisms of antibacterial ASOs.

**IMPORTANCE:**

The rise of antimicrobial resistance presents a major global health challenge. If not addressed, the death toll from resistant infections is expected to rise dramatically in the coming years. As a result, it is essential to explore alternative antimicrobial therapies. One promising approach is to target bacterial mRNAs using antisense oligomers (ASOs) to silence genes involved in essential functions, virulence, or resistance. However, delivering ASOs across bacterial membranes remains a major challenge and effective methods to monitor their uptake are limited. In this study, we develop a reporter assay to facilitate the high-throughput discovery of bacterial ASO carriers. This research paves the way for developing novel precision antisense-based antibacterial therapies.

## INTRODUCTION

Antimicrobial resistance is one of the major current global health challenges, emphasizing the need for novel strategies to treat resistant infections (1). Programmable species-specific RNA-based antibacterials are one promising alternative to conventional antibiotics (2–4). They are based on antisense oligomers (ASOs), i.e., short, single-stranded synthetic nucleic acid mimics designed to bind to complementary sequences in target mRNAs to repress their translation (5). By targeting mRNAs of essential genes, ASOs can lead to bacterial cell death (6). In addition, ASOs can be designed to suppress antibiotic resistance genes to reinstate the activity of antibiotics (7–11). Their use has been validated for multiple bacterial species, which include *Escherichia*, *Klebsiella*, *Pseudomonas*, *Salmonella,* and *Staphylococcus* species (12–18) and additional microbes (8, 19–21).

Currently, antibacterial ASOs are mostly based on peptide nucleic acid (PNA) or phosphorodiamidate morpholino (PMO) backbones due to their neutral charge, which is crucial for the traversal of the negatively charged bacterial envelope (22). Nevertheless, ASOs alone are unable to translocate into the bacterial cytoplasm and need to be coupled to a carrier (2, 4). Common carriers include cell-penetrating peptides (CPPs), which are often short, cationic, and amphiphilic peptides (23) that translocate across bacterial membranes using poorly understood mechanisms. While successful CPP-ASO conjugates have been reported for different bacterial species (8, 12–16, 19–21), our understanding of carrier properties required for optimal ASO delivery is limited.

To tackle this question, methods to assess the delivery potential of ASO carriers are required. Current methods either directly quantify CPP or ASO internalization or use indirect measurements of ASO-mediated gene regulation. To directly assess the uptake of the carrier module, CPPs can be labeled with fluorophores such as TAMRA (5-carboxytetramethylrhodamine) to monitor their localization using flow cytometry and confocal microscopy (24, 25). Nevertheless, since the delivery potential of CPPs for cargos of different sizes and physicochemical properties varies (26) and ASOs are much larger than the TAMRA dye, this assay might not accurately measure PNA delivery. Instead, mass spectrometry has been used to directly detect ASOs in the bacterial cytoplasm or periplasm (22, 27). However, mass spectrometry-based methods are relatively laborious and limited in throughput. To assess ASO activity indirectly, bacterial growth inhibition can be used as a proxy if the ASO targets an essential gene. However, antibacterial activity is not a strict read-out for effective ASO delivery because the CPP moiety itself may also be toxic (12, 13, 24). Monitoring phenotypic changes post CPP-ASO treatment is another option to evaluate the delivery potential of carriers. For example, inhibition of the *ftsZ* gene encoding an important cell division protein causes bacterial filamentation (28), which can easily be scored under the microscope. Alternatively, targeting the mRNAs of reporter proteins such as beta-galactosidase (encoded by the *lacZ* gene) (29), luciferase (30), or fluorescence proteins (31) can be used. These reporter assays have the advantage of being non-destructive, but the loss of gene expression can also result from non-specific toxicity of the CPP-ASO conjugates.

**Fig 1:**
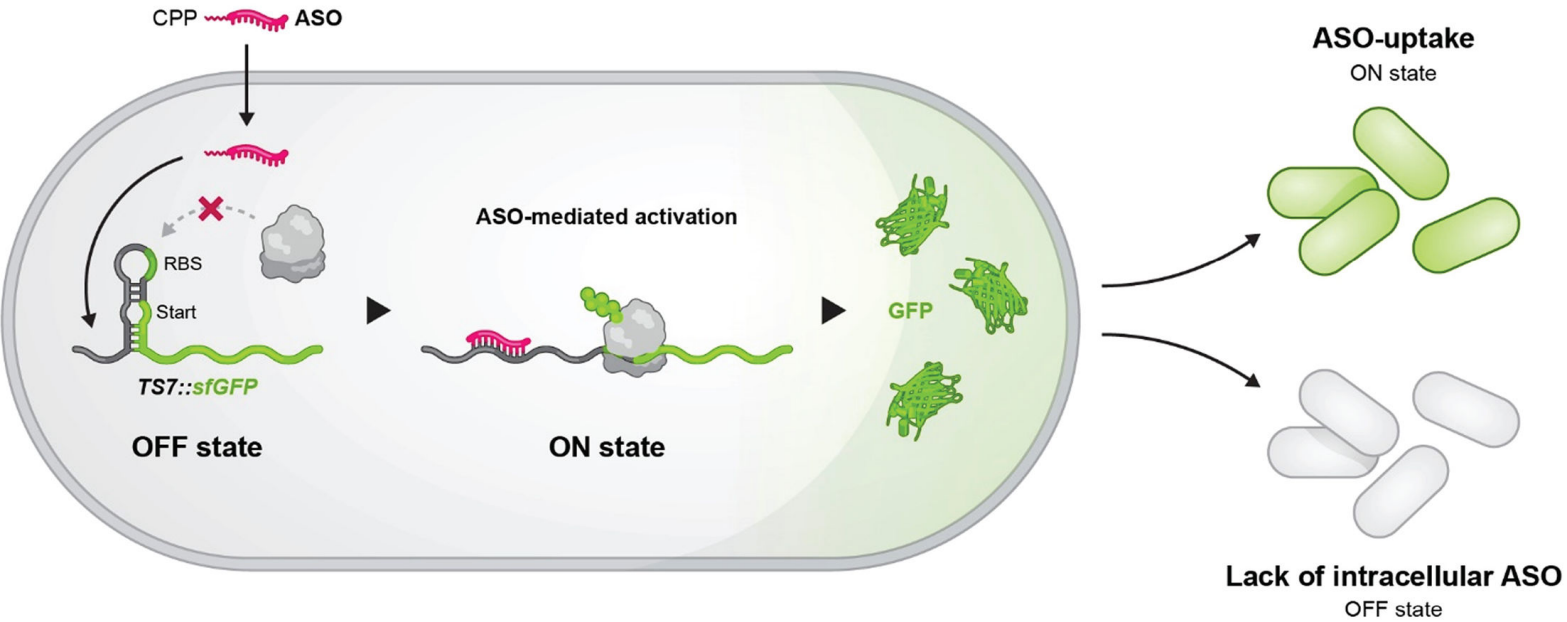
Design of the antisense activation-based “switch-on” reporter assay. Schematic overview of antisense oligonucleotide (ASO)-based RNA toehold switch activation assay. In the OFF state, the TS7 toehold switch sequesters the ribosome-binding site (RBS) and the start codon, thus preventing translation of the downstream reporter gene encoding superfolder GFP (sfGFP). Upon ASO uptake, the ASO hybridizes with the stem-loop of TS7 and opens the inhibitory secondary structure. The RBS becomes accessible, and reporter protein translation is initiated (ON state). In case of inefficient CPP-PNA delivery, the reporter will remain in its off-state. CPP, cell-penetrating peptide.

Here, we have developed a translational “switch on” reporter assay to assess ASO delivery into the bacterial cytoplasm. It draws inspiration from a common post-transcriptional mechanism of gene activation by small regulatory RNAs (sRNAs) in bacteria, i.e., sRNA-mediated disruption of an inhibitory RNA structure around the ribosome binding site (RBS) of a target mRNA (32). To design our reporter, we chose an established synthetic RNA toehold switch (33), referred to as TS7 in this report, that sequesters the RBS and start codon of the mRNA of a fluorescent protein in a stem-loop structure (Fig. 1). The switch can be activated by co-expression of antisense RNAs that bind the 5′ flank of the stem-loop (33). We designed PNA-based ASOs that similarly bind the switch and activate the synthesis of the fluorescent reporter protein, resulting in an “on-state.” Variation in fluorescence resulting from this “on-state” can be used to infer the efficacy of CPPs to deliver ASOs into bacteria, independent of their antibacterial activity. We tested this reporter in different bacterial strains using variable ASO concentrations, growth media, and fluorescent reporter proteins. We also successfully conducted a small screen of 10 CPPs. This sets the stage for high-throughput screens for new ASO carriers, be these short peptides or other delivery vehicles for traversal of the bacterial envelope.

## RESULTS

### PNA-mediated activation of the RNA toehold switch *in vitro*

**Fig 2:**
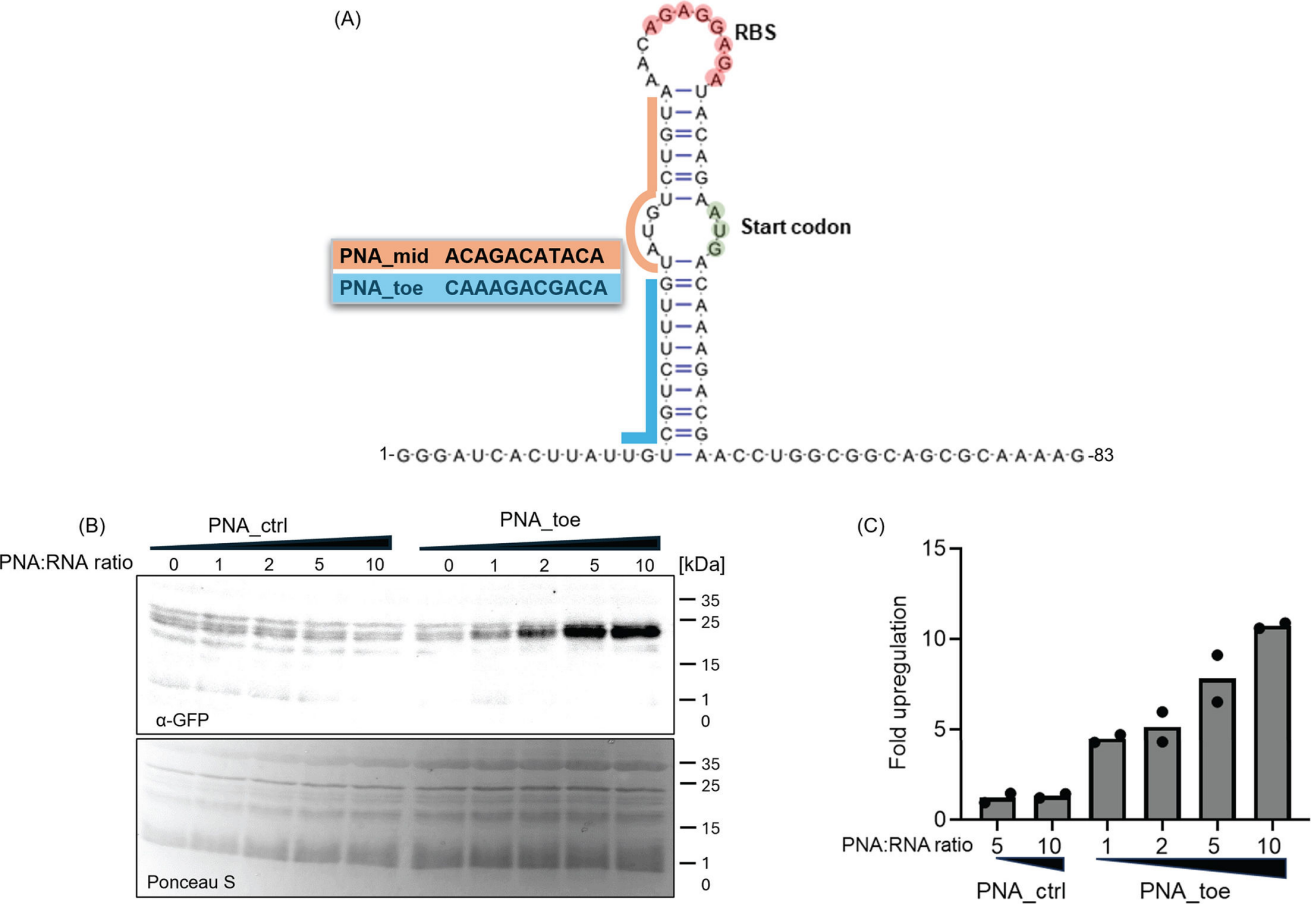
Antisense-mediated activation of toehold switch-controlled GFP expression *in vitro*. (**A**) Structure of the TS7 toehold switch. The RBS and translational start codon are highlighted in red and green, respectively. The PNA sequences with their respective target sites are indicated in blue and orange. (**B and C**) *In vitro* transcribed *TS7::gfp* RNA was subjected to an *in vitro* translation assay. Synthetic *TS7::gfp* fusion mRNA was incubated with the indicated molar excesses of a non-targeting PNA_ctrl or PNA_toe. As a negative control, an equal volume of water was added to the samples (“0”). RNA was translated *in vitro,* and samples were separated on 12% SDS-PAA gels with subsequent blotting on nitrocellulose membranes. (**B**) Western blot-based detection of GFP was performed using a monoclonal α-GFP antibody (upper panel). PonceauS staining was used to control for equal loading (lower panel). (**C**) Changes in GFP protein levels were quantified using ImageJ. Relative protein expression levels of GFP were calculated based on the water control sample. Bars represent the mean of two independent experiments; individual data points are shown as well.

We build the ASO sensor component of our switch-on reporter upon the TS7 toehold switch, an 83 bp sequence, which, when transcribed, can form an imperfect 18 bp long RNA hairpin (Fig. 2A), as reported by Green et al. (33). In the original description, TS7 was activated by the co-expression of a *trans*-acting antisense RNA with 30 nucleotides complementarity to the 5′ untranslated region of the toehold switch (33). We hypothesized that due to the higher affinity of PNA to RNA (34), shorter PNAs in the typical length range of antibacterial ASOs could achieve a similar effect. To test if 11mer PNAs can activate the toehold switch, we first performed an *in vitro* translation assay. We titrated *in vitro* transcribed *TS7::gfp* RNA with varying concentrations of a PNA that targets the toehold region of the TS7 switch (PNA_toe) (Fig. 2A). Accumulation of GFP after *in vitro* translation was detected by western blot (Fig. 2B). The addition of PNA_toe led to a dose-dependent increase in GFP levels, reaching ~11-fold upregulation at 10× molar excess of PNA over RNA compared to the water control (Fig. 2C). To control for non-specific activation by PNA, we used a non-targeting PNA (PNA_ctrl), which had no effect even when provided at a 10× molar excess over *TS7::gfp* RNA. This *in vitro* assay demonstrates that the 11mer PNA_toe can activate the TS7 RNA toehold switch for the synthesis of the fused reporter protein.

### Activation of the toehold switch upon PNA delivery *in vivo*

**Fig 3:**
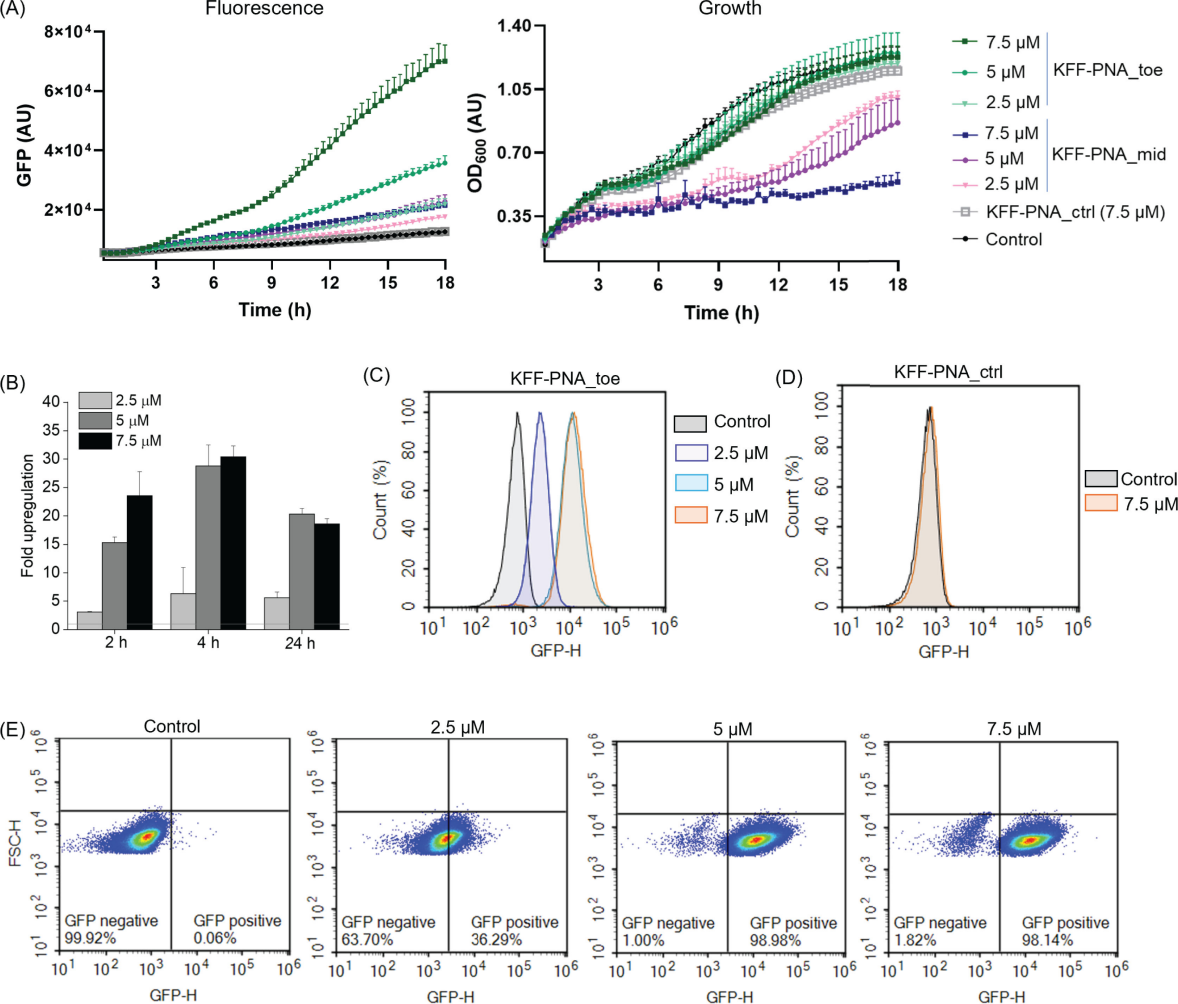
KFF-PNA-mediated activation of toehold switch-controlled sfGFP expression in *Salmonella*. *Salmonella* carrying the *TS7::sfgfp* encoding plasmid were treated with increasing concentrations of KFF-PNA_toe or KFF-PNA_mid. As negative controls, a non-targeting PNA (KFF-PNA_ctrl) or water (Control) was used. (**A**) Fluorescence (sfGFP) (left) and bacterial growth (right) were recorded in a microplate reader for 18 h post-PNA treatment. Data points indicate mean sfGFP fluorescence intensities (left panel) and the optical densities at 600 nm (OD_600_; right panel). Error bars show the standard deviation of the mean fluorescence and OD from two replicates. (**B–E**) Flow cytometric analysis of sfGFP induction post PNA treatment. (**B**) Concentration-dependent sfGFP induction after treatment with KFF-PNA_toe for 2 h, 4 h, and 24 h, respectively. Bars indicate the relative upregulation of PNA-treated samples compared to the water-treated control. Upregulation was calculated based on the median fluorescence intensities. Error bars indicate the standard deviation of median fluorescence intensity from two independent experiments. (**C, D**) Flow cytometry histograms showing changes in sfGFP fluorescence 4 h post-treatment with (**C**) KFF-PNA_toe at 7.5 µM, 5 µM, and 2.5 µM or (**D**) a non-targeting PNA (KFF-PNA_ctrl) at 7.5 µM. (**E**) Density plots of sfGFP induction in bacterial populations post-treatment with KFF-PNA_toe at the indicated concentrations. For the control (left panel), an equal volume of water was added. AU indicates arbitrary units. Each experiment was performed at least two times.

Next, we tested whether 11-mer PNAs can activate the toehold switch in bacteria. We established the reporter assay in *Salmonella enterica serovar Typhimurium* (referred to as *Salmonella* hereafter), which we have previously used as a model organism for studying PNAs (13, 17, 22). In constructing a reporter plasmid, we cloned the TS7 sequence upstream of the coding sequence of superfolder GFP (sfGFP) and downstream of a constitutive P_LtetO-1_ promoter in the pXG10-SF vector (35) (see Materials and Methods for details). The final product is a fusion protein (TS7::sfGFP) in which the 10 amino acids from the short TS7 coding sequence are genetically linked to the N-terminus of sfGFP. We introduced the reporter plasmid in *Salmonella*. The bacteria were grown to an early log-phase in standard Mueller-Hinton broth (MHB), commonly used for testing antibacterial activity (36). As a proof-of-concept, we chose to deliver the PNAs through conjugation with (KFF)_3_K, a frequently used CPP (referred to as KFF hereafter). We tested two PNAs, PNA_toe as used above *in vitro* and a PNA that targets the mid-stem region (PNA_mid) of the switch (Fig. 2A). We treated the bacterial culture with varying concentrations (2.5 µM, 5 µM, or 7.5 µM) of the KFF-PNAs in a microtiter plate, recording GFP fluorescence and turbidity as bacterial growth continued. We did not use higher concentrations to avoid nonspecific side effects. While treatment with KFF-PNA_ctrl or water caused no substantial increase in fluorescence over time, both PNA_toe and PNA_mid led to a concentration-dependent increase in fluorescence, with PNA_toe being more effective (Fig. 3; Fig.S1). Moreover, PNA_toe had no effect on bacterial growth, whereas PNA_mid impaired bacterial growth (Fig. 3A). Based on both, its superior ability to activate the reporter and its lower toxicity, we selected PNA_toe as the activating ASO moiety for further experiments.

In contrast to plate reader-based measurements, which provide values of the population average, flow cytometry offers a more quantitative readout with single-cell measurements (37). Using flow cytometry, we validated the observations from the microplate reader, confirming both the concentration-dependent and time-dependent variation in GFP fluorescence after PNA treatment (Fig. 3B and C; Fig. S2). Interestingly, a decrease in the fluorescence was observed at 24 h relative to 4 h post-treatment, potentially due to degradation of the KFF moiety in the medium (27), which renders the remaining PNA constructs unable to enter the bacterial cells. As expected, KFF-PNA_ctrl did not activate GFP expression (Fig. 3D). The flow cytometry density plots revealed a gradual homogeneous increase in the percentage of the GFP positive population with increasing PNA concentration (Fig. 3E). The dynamic range of the flow cytometry readout (determined as the highest upregulation observed with the KFF-PNA compared to water-treated control) exceeds that of the microplate reader (1.5–30 vs 1.5–10, respectively). Since treatment with either 7.5 µM or 5 µM KFF-PNA_toe showed equally strong activation of the reporter 4 h post-treatment, we settled on 5 µM for all further experiments to minimize potential toxicity.

### An improved toehold switch enhances PNA-mediated activation

**Fig 4:**
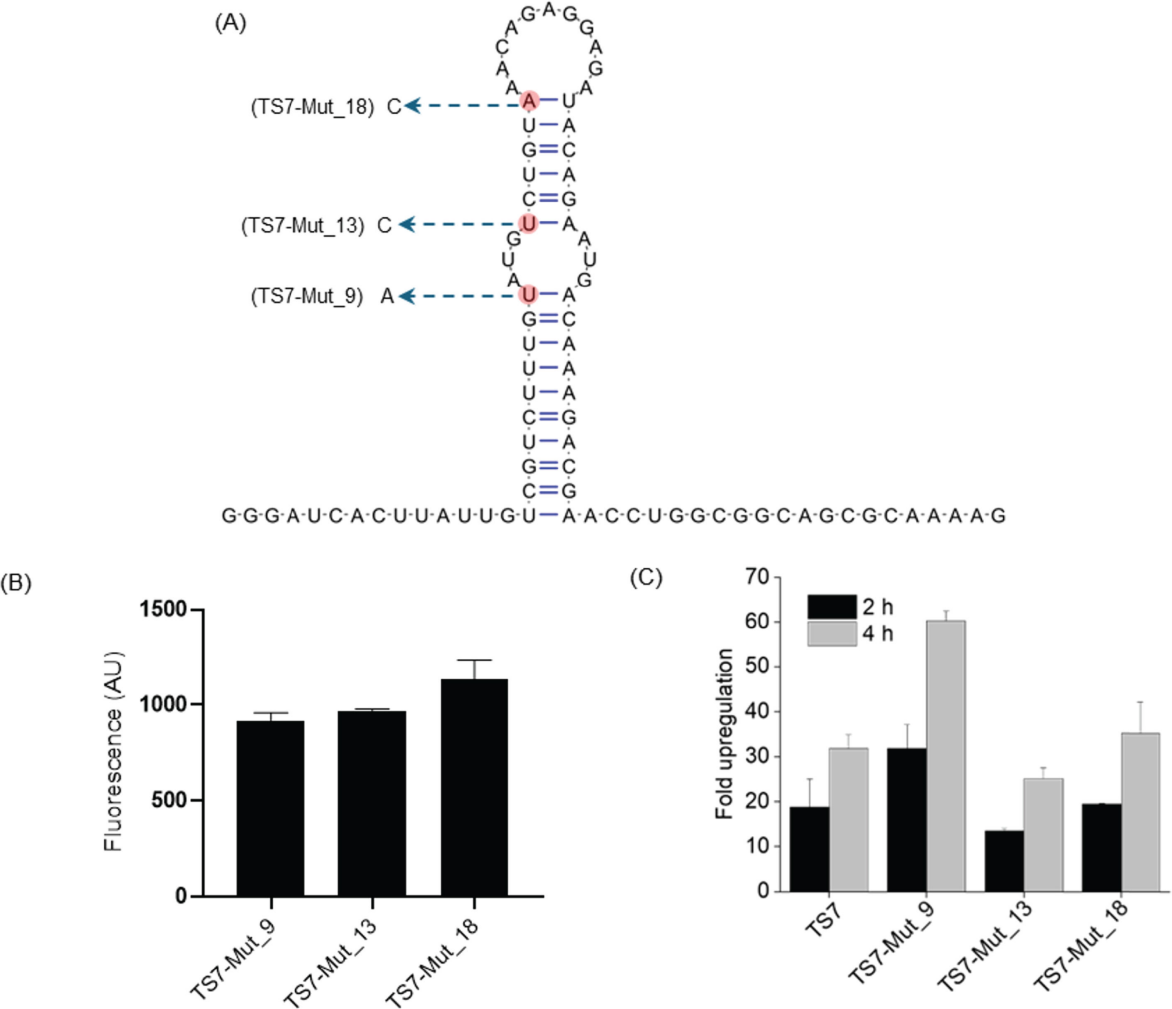
Impact of single-nucleotide mutations in the stem of the toehold switch (TS7) on the reporter’s sensitivity**.** (**A**) Point mutations (indicated in red) introduced within the *TS7::sfGFP* toehold switch to improve response to PNA-mediated activation. (**B, C**) Flow cytometric analysis of *Salmonella* bearing plasmids containing the original or mutated *TS7::sfGFP* reporter constructs. (**B**) Comparative representation of basal fluorescence intensities of mutated and original toehold switch constructs measured by flow cytometry. (**C**) Bacterial cells containing the different *TS7::sfGFP* constructs were treated with 5 µM of KFF-PNA_toe, and sfGFP signals were measured at 2 h and 4 h post-treatment. Bars indicate relative upregulation of sfGFP calculated based on the median fluorescence intensities of PNA-treated samples relative to the water control. Error bars indicate the standard deviation calculated from two independent experiments.

Our activation assay is based on the ability of an ASO to either open or prevent the formation of the inhibitory stem-loop of the RNA toehold switch to initiate mRNA translation . We reasoned that we might be able to improve the dynamic range and sensitivity of the assay by weakening the stem-loop for easier melting. To this end, we mutated positions 9, 13, and 18 of the stem, all of which are outside the loop and binding site of PNA_toe (Fig. 4A). These mutations caused a slight increase in basal reporter protein synthesis as seen by higher fluorescence without PNA treatment (Fig. 4B). Seeking the highest signal-to-background ratio post-PNA treatment, we treated cells carrying these mutated switch variants with KFF-PNA_toe for 4 h, the time point at which we previously observed the highest activation of the reporter (Fig. 3B). While KFF-PNA_toe resulted in around 30-fold upregulation of the reporter with the original sequence (Fig. 3B), we observed a ~50- to 60-fold activation of the switch mutated at position 9 (TS7-Mut_9, Fig. 4C). By contrast, mutations at positions 13 (TS7-Mut_13) or 18 (TS7-Mut_18) led to no improvement over the original switch. We also examined fluorescence 2 h after treatment to assess if the mutations facilitated faster activation, but this was not the case (Fig. 4C). Since TS7-Mut_9 offered the highest dynamic range, we selected this reporter for the experiments below.

### Influence of growth medium and fluorescent proteins on reporter activation

**Fig 5:**
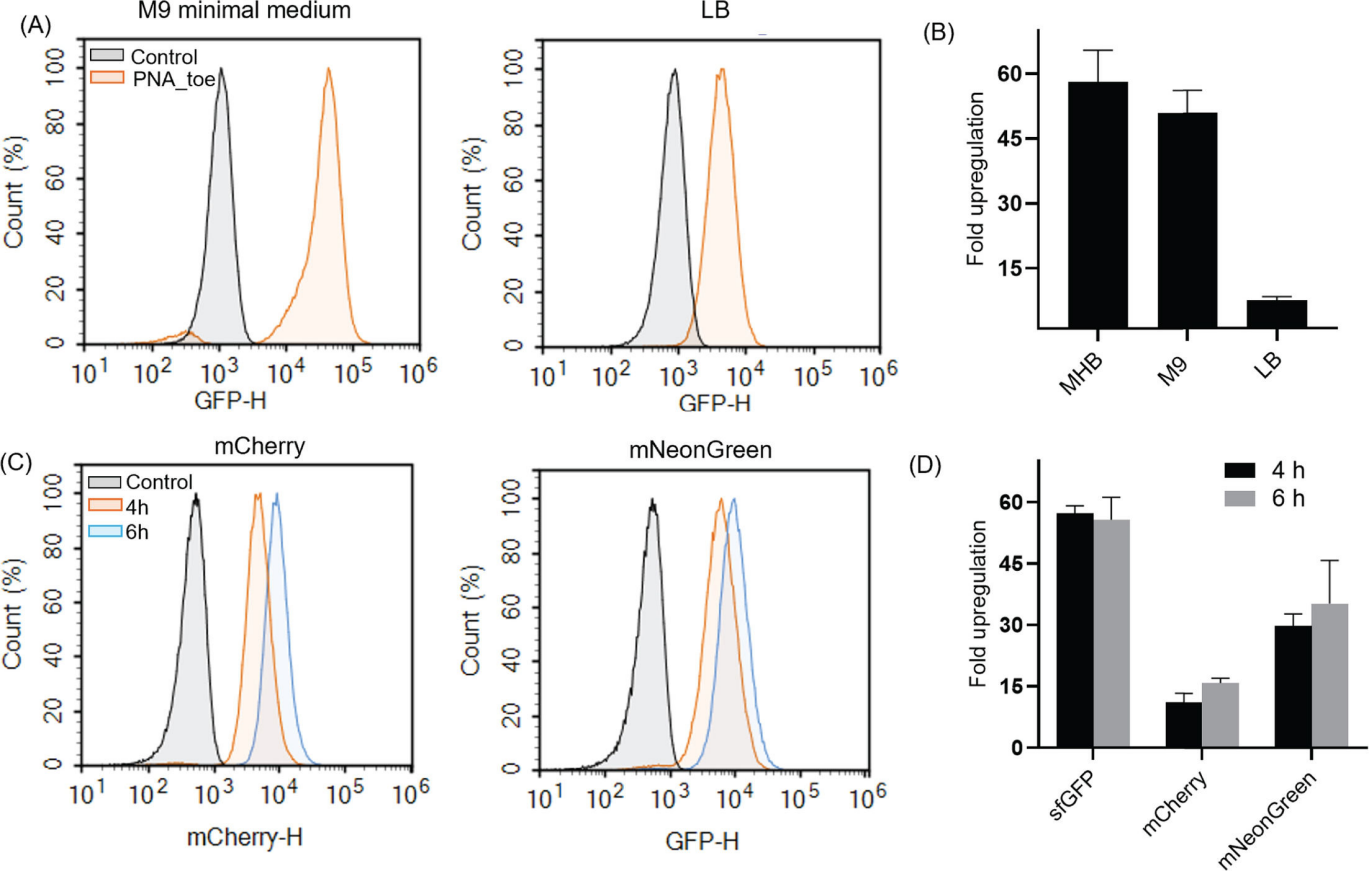
Effect of culture media and fluorescent reporters on the degree of PNA-mediated toehold switch activation in S*almonella*. (**A**) Flow cytometry histograms of sfGFP fluorescence of *Salmonella* containing the *TS7::sfGFP-Mut_9* reporter construct treated with KFF-PNA_toe at 5 µM in M9 minimal medium (left) or Luria (LB) broth (right) for 4 h. Control indicates bacteria treated with an equal volume of water. (**B**) Fold upregulation of sfGFP fluorescence in different culture media at 4 h post-treatment relative to water-treated control cells. (**C**) Flow-cytometry histograms of fluorescence of *Salmonella* containing the *TS7-Mut_9::mCherry* (left) or *TS7-Mut_9::mNeonGreen* (right) reporter constructs, treated with KFF-PNA_toe at 5 µM in Mueller Hinton medium for 4 h and 6 h. (**D**) Fold upregulation of mCherry or mNeonGreen fluorescence post-treatment with KFF-PNA_toe at 5 µM. (**B, D**) Fold upregulation of fluorescence was calculated based on the median fluorescence intensities of PNA-treated samples relative to the water-treated control, measured by flow cytometry. Error bars indicate the standard deviation calculated from two independent experiments.

The composition of the culture media can influence the interaction between cationic CPPs and bacterial cells (38, 39) and so might affect CPP-mediated PNA delivery. To study how different media affected CPP-PNA-mediated reporter activation, we compared the upregulation of sfGFP expression after 4 h treatment in three different standard culture media: MHB (used above), a minimal medium referred to as M9 and nutrient-rich Luria broth (LB). In both MHB and M9, KFF-PNA_toe triggered a ~50- to 60-fold activation of the reporter (Fig. 5A and B). In contrast, we observed only ~6- to 8-fold upregulation in LB. This shows that the activity of the reporter assay depends on the culture medium. Given that MHB is commonly used for testing antimicrobials, we continued with MHB for all subsequent studies.

To assess how the reporter protein influences the dynamic range of the assay, we tested two additional commonly used fluorescent proteins, mNeonGreen and mCherry. In accordance with the fact that sfGFP is among the brightest fluorescent proteins and characterized by a rapid maturation time in Enterobacteriaceae (40), the sfGFP-based reporter showed the highest upregulation after 4 h and 6 h (Fig. 5D). Both mCherry and mNeonGreen showed a lower fold activation than sfGFP at both time points, potentially due to longer maturation times and poor stability, at least for mCherry (41). Since sfGFP showed higher and faster upregulation than both mCherry and mNeonGreen, we continued with the sfGFP reporter.

### The toehold switch reporter can provide insights into CPP-PNA uptake mechanisms

**Fig 6:**
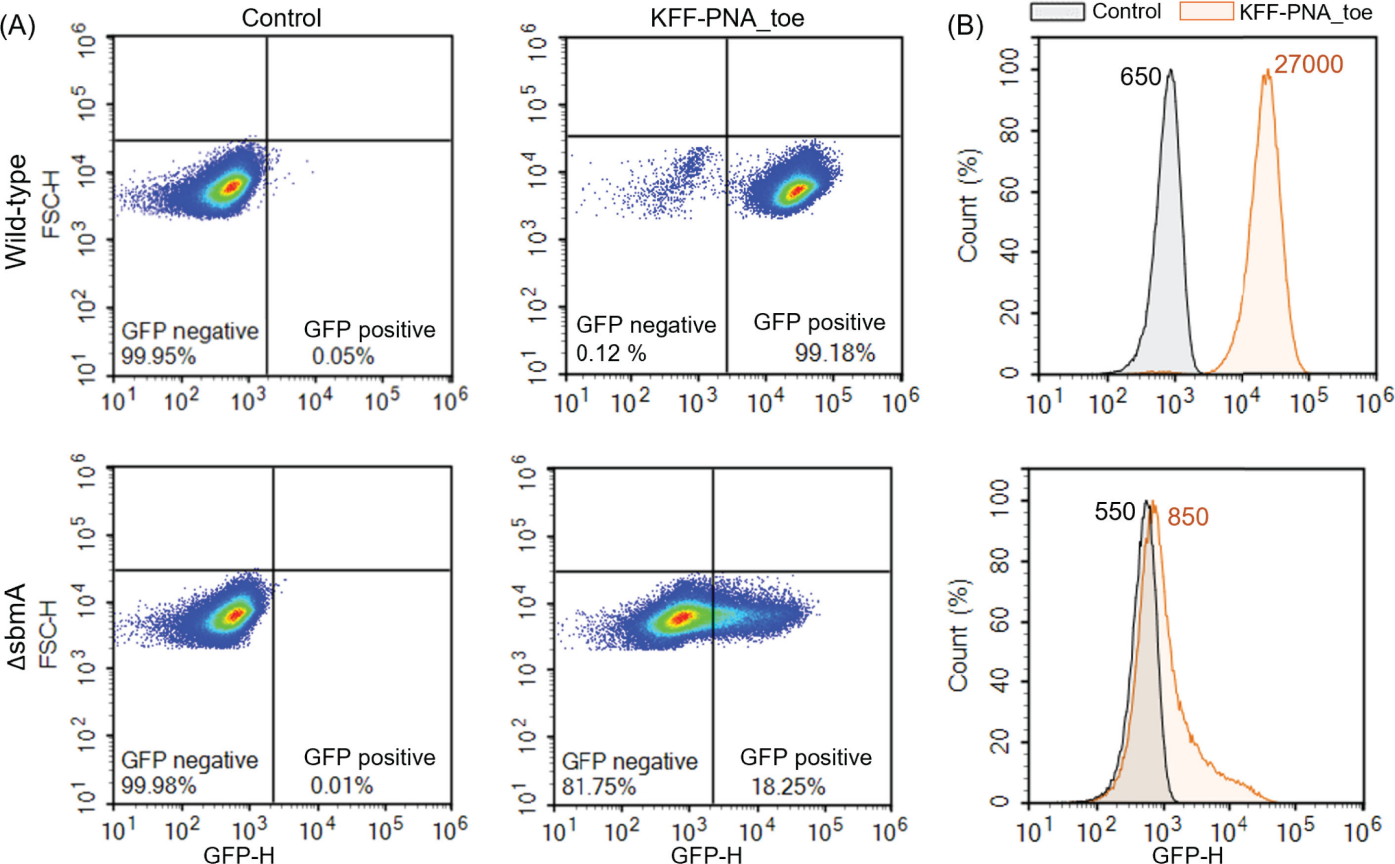
Flow cytometric analysis of CPP-PNA uptake using the toehold switch reporter in a Δ*sbmA* mutant of *Salmonella***.** Flow cytometric (**A**) density plots and (**B**) histograms depicting the heterogeneity in sfGFP expression upon activation of *TS7-Mut_9::sfGFP* 4 h post-treatment with KFF-PNA_toe (5 µM). The upper panels show results for *Salmonella* wild-type, and the lower panels depict the results for an isogenic Δ*sbmA* mutant. Control indicates bacteria treated with an equal volume of water. (**B**) Median fluorescence intensity values for water-treated control (Control, black) and PNA-treated (KFF-PNA_toe, orange) samples are given in the plots.

To confirm that activation of the toehold-switch reporter correlates with successful uptake of PNAs, we used a *Salmonella* Δ*sbmA* mutant strain unable to express the inner membrane transporter SbmA. This protein has been shown to mediate the uptake of KFF-conjugated PNAs into the taxonomically close relative, *E. coli* (27). In the Δ*sbmA* strain, treatment with KFF-PNA_toe triggered only marginal GFP expression in a small subpopulation of cells (ca 20%) as opposed to nearly 100% of all cells in the corresponding wild-type strain (Fig. 6A). The overall fluorescence intensity (FI) of the Δs*bmA* mutant was also much lower than the wild-type strain (Fig. 6B). These results validate that the assay reports PNA uptake and indicate that the reporter can also be used to assess the involvement of bacterial transporters in PNA delivery.

### The toehold switch reporter enables the assessment of CPP-mediated PNA delivery

**Fig 7:**
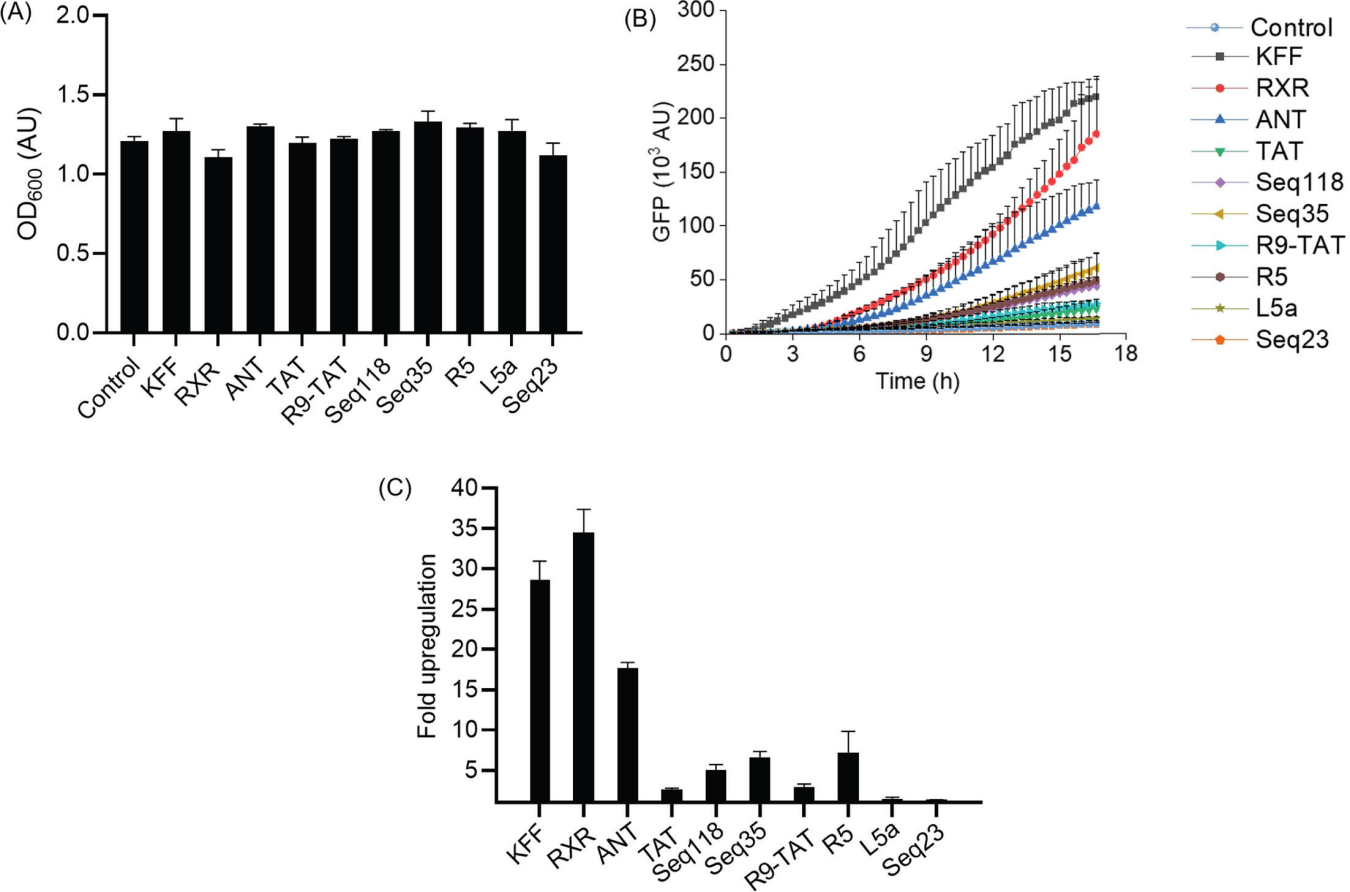
Antisense activation of toehold switch reporter enables screening of CPPs for PNA delivery. (**A–C**) *Salmonella* carrying the *TS7-Mut_9::sfGFP* encoding plasmid were treated with different CPP-PNA_toe constructs at 5 µM. As a negative control, an equal volume of water (Control) was added instead. (**A**) Bacterial growth (OD_600_) and (**B**) sfGFP fluorescence (GFP) were recorded in a microplate reader for 17 h post-PNA treatment. AU, arbitrary units. (**C**) Relative upregulation of sfGFP fluorescence was calculated based on the fluorescence intensities at 17 h of PNA-treated samples relative to the water control. Error bars indicate the standard deviation of fold upregulation calculated from two independent experiments.

Having established that the reporter assay reads out PNA delivery, we applied it to assess new CPP candidates. We tested a small library of 10 CPPs for their ability to deliver PNA_toe into *Salmonella*. We included the established CPPs KFF, RXR, TAT, and ANT, all of which are known to deliver PNA into *Salmonella* (13). We also included six other CPPs with varying cationicity and length and with reported applications in prokaryotic or eukaryotic cells (25, 42) from our in-house collection (Table 1). CPP-PNA conjugates differ in their secondary structure and physicochemical properties from unconjugated CPPs (43) and, therefore, behave differently. For example, we previously observed that RXR- and TAT-PNA_ctrl conjugates are more toxic than the unconjugated CPPs (13). Therefore, we used CPPs conjugated to the non-targeting PNA_ctrl as controls. None of these PNA_ctrl conjugates activated the reporter Table S1 and S2). In addition to fluorescence, we simultaneously measured bacterial growth, with potential growth impairment flagging toxicity of the CPP moiety. Although there were no strong toxic effects, we did observe mild growth retardation for RXR and Seq23 (Fig. 7A). To control for these effects, fluorescence was normalized to the optical density of the bacterial culture (Fig. S3). We observed a rapid increase in fluorescence signal for the PNA_toe conjugates of KFF and RXR, while the other CPPs showed a slower increase (Fig. 7B). At 17 h post-treatment, KFF, RXR, and ANT exhibited the highest fold increase in fluorescence compared to the water-treated control, followed by R5, Seq118, and Seq35 (Fig. 7C). A negligible upregulation was observed with TAT, R9-TAT, and L5a, and none with Seq23. These data demonstrate that our reporter assay can be used to screen CPP candidates by using the microplate reader.

**TABLE 1:** Description of cell-penetrating peptides (CPP) used in this study

CPP	Sequence^a^	Number of	
		Cationic amino acids	Hydrophobic groups
KFF	KFFKFFKFFK	4	6
RXR	RXRRXRRXRRXRXB	8	6
ANT	RQIKIWFQNRRMKWKK	7	6
TAT	GRKKRRQRRRYK	9	1
R9-TAT	GRRRRRRRRRPPQ	9	0
Seq118	RRRQRRKKRGY	8	1
Seq35	RKKRRQRR	7	0
R5	RRRRR	5	0
L5a	RRWQW	2	2
Seq23	VALLPAVLLA	0	9

^a^ CPP sequences are shown from N to C termini. X indicates 6-amino-hexanoic acid; B is β-alanine.

**Fig 8:**
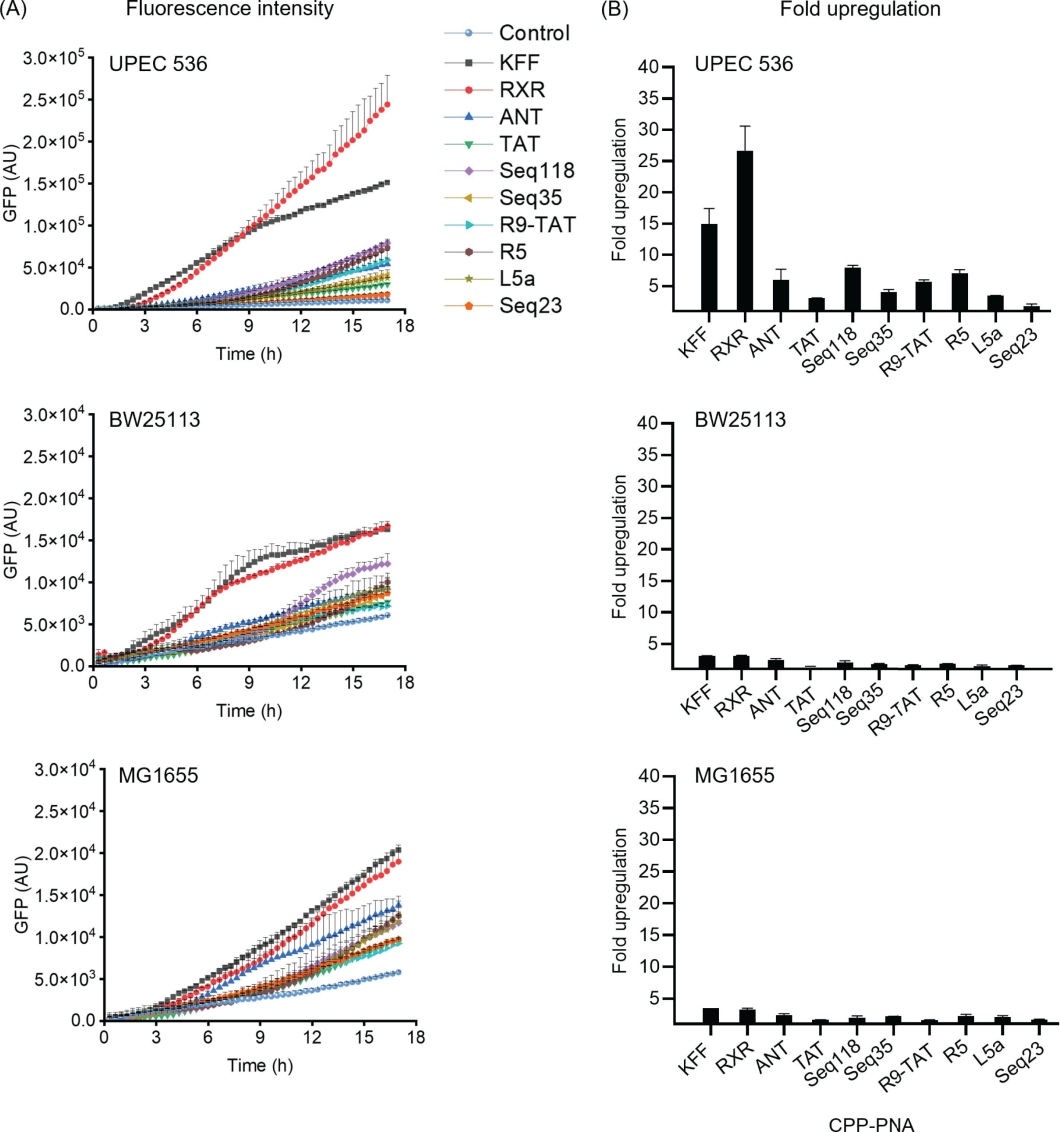
Examining the performance of the toehold switch assay for screening of CPPs against *E. coli*. Various *E. coli* strains carrying the *TS7-Mut_9::sfGFP* encoding plasmid were treated with different CPP-PNA_toe constructs at 5 µM. As negative control, an equal volume of water (Control) was added instead. (**A**) Fluorescence intensity of sfGFP was recorded in a microplate reader for 17 h post-treatment measured in a microplate reader. (**B**) Upregulation in sfGFP fluorescence calculated relative to the water control based on the normalized fluorescence intensities at 17 h post-treatment. The experiment was performed two times, and bars indicate the mean value; error bars indicate the standard deviation from two independent experiments.

Having established the CPP screening assay in *Salmonella*, we next tested it in *E. coli*. We chose the *E. coli* K12 strains BW25113 and MG1655, and the uropathogenic isolate *E. coli* 536 (UPEC 536). Using the same reporter (*TS7-Mut_9::sfGFP*) and conditions as above, we observed the highest reporter activation for KFF and RXR conjugates in all these three *E. coli* strains similar to *Salmonella* (Fig. 8). Seq23 remained the least effective PNA carrier. Importantly, *E. coli* BW25113 and MG1655 displayed ~10-fold lower fluorescence signals for all CPPs compared to UPEC 536 despite similar basal fluorescence in all strains (Fig. 8). To test if the low fluorescence was due to lower sfGFP protein levels, we performed a western blot analysis after treating the bacteria with CPP-PNAs (Fig. S4). The upregulation of sfGFP protein correlated with fluorescence upregulation. The *E. coli* K12 strains were less susceptible than *Salmonella*. For these strains, a more sensitive method compared to the microplate reader such as flow cytometry might provide better results.

### Flow cytometry shows dynamic differences in toehold switch activation by CPP-PNAs

**Fig 9:**
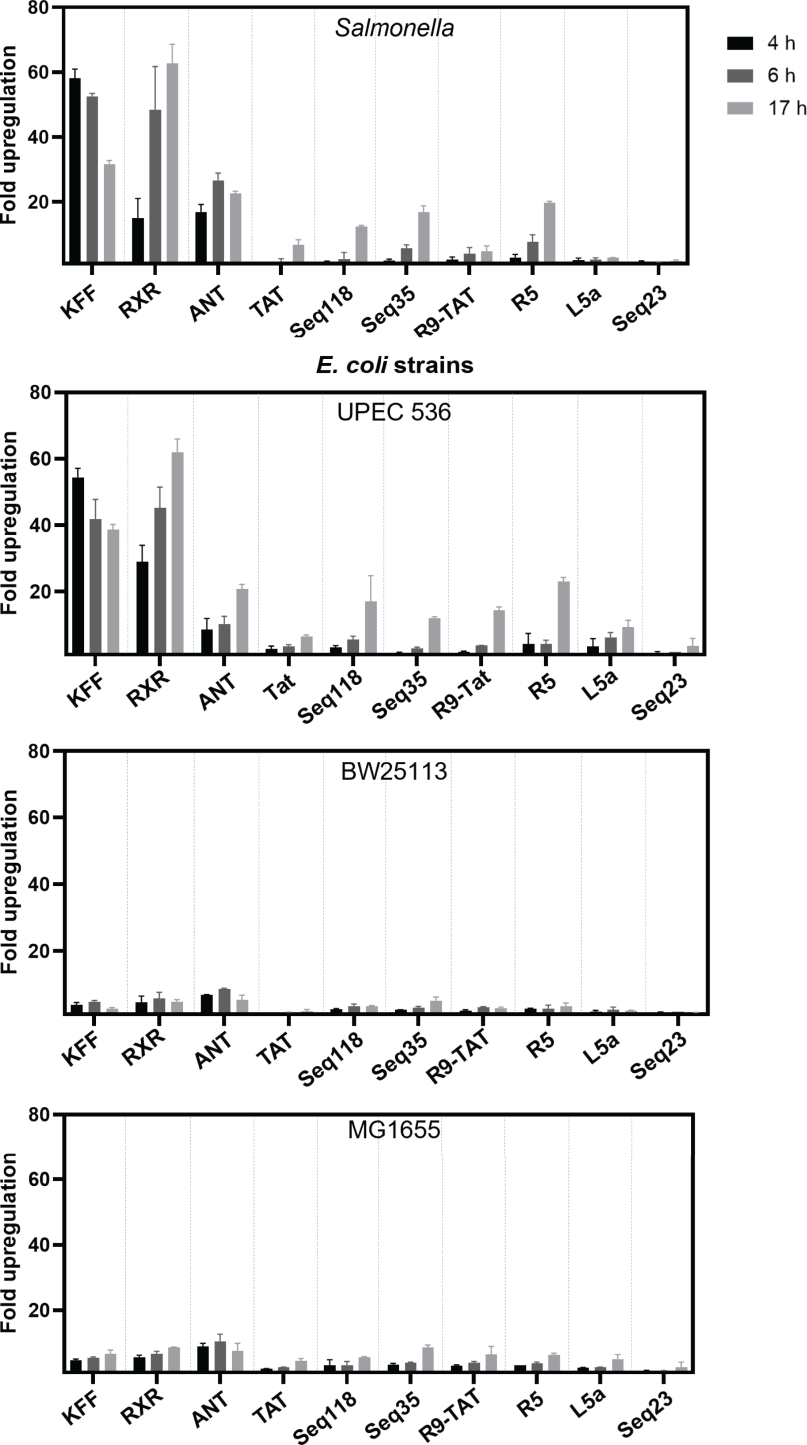
Flow cytometric analysis of fluorescence of sfGFP informs on delivery efficiency of CPPs. Bacterial cultures expressing the *TS7-Mut_9::sfGFP* reporter construct treated with the indicated CPP-PNA_toe constructs at 5 µM each. The median fluorescence intensity of sfGFP was measured after 4 h (black bars), 6 h (dark gray bars), and 17 h (light gray bars) of treatment using flow cytometry. Relative upregulation of sfGFP is shown and was calculated by dividing the median fluorescence intensity of the test samples by the median fluorescence intensity of the control samples, to which water was added. Error bars indicate the standard deviation calculated from two independent experiments.

**Fig 10:**
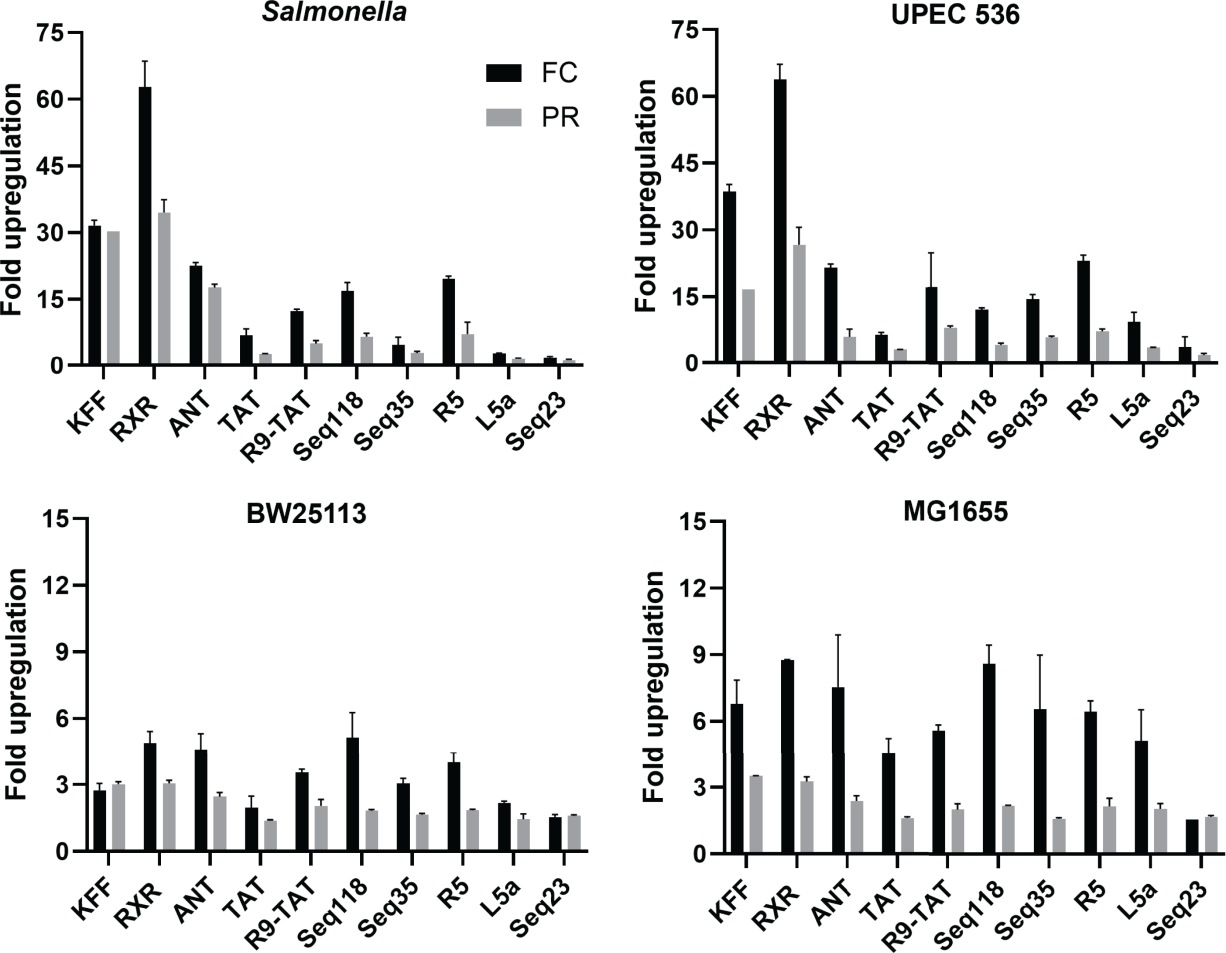
Comparison of CPP-PNA_toe-mediated reporter activation evaluated by flow cytometry (FC) versus microplate reader (PR). Average fold upregulation in sfGFP fluorescence was calculated relative to the respective water-treated controls. Fluorescence was measured 17 h post-treatment with various CPP-PNA_toe constructs using the microplate reader (gray bars; see also Fig. 8) or flow cytometry (black bars; see also Fig. 9). Error bars indicate the standard deviation of fold upregulation from two independent experiments.

According to previous RNA-seq analyses (12, 13, 22), CPP-PNAs can strongly downregulate target mRNAs within 15 min. This suggests that reporter activation could possibly be scored earlier, permitting more sensitive detection of GFP fluorescence. We therefore repeated the screen conducted and assayed fluorescence by flow cytometry at 4, 6, and 17 h post-treatment. In all strains, KFF, RXR, and ANT showed rapid upregulation at 4 h, whereas Seq35, Seq118, R9-TAT, and R5 only showed upregulation at the 17 h time point (Fig. 9); Seq23 consistently was the least active carrier. The level of upregulation observed in the *E. coli* K12 strains was again substantially lower than in *Salmonella* and UPEC 536. Nevertheless, as compared to the plate reader, flow cytometry showed a higher dynamic range and sensitivity to study the efficacy of CPP-mediated ASO delivery in all the tested bacteria (Fig. 10). Interestingly, we observe CPP-dependent differences in the dynamics of ASO-mediated sfGFP induction. For example, while fluorescence increases over time for most CPPs, KFF showed a decrease (Fig. 9). This might be related to the stability of KFF in the culture media. Overall, our results show that CPP-mediated PNA delivery varies over time and in different bacterial strains.

### Reporter activation correlates with the activity of antibacterial CPP-PNA conjugates

**Fig 11:**
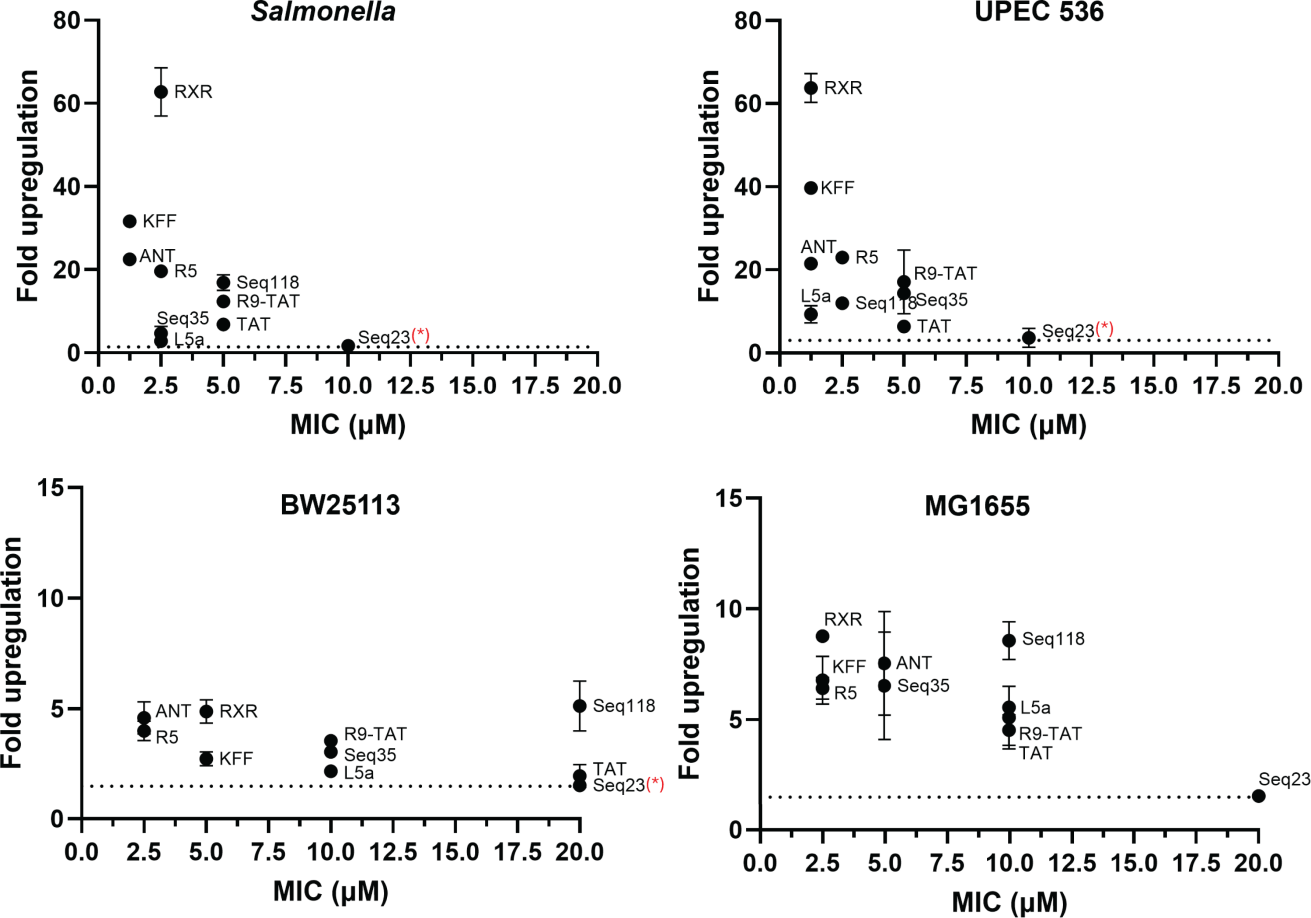
Comparison of CPP-PNA_toe-mediated activation with the growth inhibitory capacity of CPP-PNA constructs targeting the essential gene *acpP*. CPP-PNA-mediated activation of the reporter assay was plotted against the growth inhibitory activity of CPP-*acpP* constructs. Average fold upregulation in sfGFP fluorescence (*y*-axis) was calculated relative to the respective water-treated controls. Fluorescence was measured 17 h post-treatment with various CPP-PNA_toe constructs using flow cytometry (shown in Fig. 9). Cognate CPP-PNA constructs targeting the mRNA of the essential gene *acpP* were used to determine their minimal inhibitory concentration (MIC) (*x*-axis). Each data point indicates the mean value of sfGFP induction and MIC, and error bars indicate the standard deviation of two independent experiments. “*” indicates that the MIC could not be determined for Seq 23 and is >10 µM in *Salmonella* and UPEC 536, >20 µM in the *E. coli* BW25113.

To assess if reporter activation correlates with antibacterial activity, we determined the minimum inhibitory concentration (MIC) of the CPPs tested above when conjugated to the antibacterial 10mer PNA sequence that targets the mRNA of *acpP* (acyl carrier protein), a well-known target of antibacterial PNAs (13, 44). We observed that RXR, KFF, and ANT, which showed rapid and marked upregulation in the reporter assay also showed low MICs (<2.5 µM against *Salmonella* and UPEC 536; <5 µM against the K12 strains) (Fig. 11; Fig. S5; Table 2). The Seq23 CPP-PNA conjugate, which showed no reporter activation, had no antibacterial activity (MIC >10 µM). In general, the two *E. coli* K12 strains (BW25113, MG1655) were less susceptible to CPP-*acpP* treatment than *Salmonella* and UPEC 536, again consistent with the reporter assays (Fig. 11). We also observed discrepancies between reporter activation and antibacterial activity. Seq118, which activated the reporter in *E. coli* BW25113 by fivefold, failed to inhibit bacterial growth of this strain. L5a barely activated the reporter in *Salmonella* (<3-fold) but mediated strong inhibition of both *Salmonella* and UPEC 536 (MIC ≤2.5 µM) (Fig. 11). Despite these inconsistencies, results of the switch-on assay generally correlate with the antibacterial assay, demonstrating potential of our reporter for identifying new carriers for antisense antibiotics.

**TABLE 2:** Antibacterial activity of CPP-*acpP* PNAs against *Salmonella* and three *E. coli* strains^*a*^

CPP-*acpP*	CPP sequence	MIC (μM)			
		*Salmonella*	UPEC 536	*E. coli* MG1655	*E. coli* BW25113
KFF	KFFKFFKFFK	1.25^*b*^	1.25^*c*^	2.5	2.5–5
RXR	RXRRXRRXRRXRXB	2.5^*b*^	1.25^*c*^	2.5	5
ANT	RQIKIWFQNRRMKWKK	1.25	1.25	5	2.5
TAT	GRKKRRQRRRYK	5^*c*^	5	10	20
R9-TAT	GRRRRRRRRRPPQ	5	5	5	10
Seq118	RRRQRRKKRGY	5	5	10	20
Seq35	RKKRRQRR	2.5	5	5–10	10
R5	RRRRR	2.5	2.5	2.5	2.5
L5a	RRWQW	2.5	1.25	10	10
Seq23	VALLPAVLLA	>10	>10	20	>20

^*a*^ All CPPs were conjugated to the PNA sequence, CTCATACTCT, antisense to the translation initiation region of the *acpP* mRNA. Bacterial suspensions were adjusted to 10^5^ cfu/mL and exposed to gradually decreasing concentrations of each CPP-PNA in MHB. The table summarizes all MICs in µM determined for each of the tested bacterial strains.

^*b*^ Data from reference 13.

^*c*^ Data from reference 12.

## DISCUSSION

Effective transport across the bacterial envelope remains a bottleneck for the application of antimicrobial ASOs, necessitating the development of new ASO carriers. To facilitate the discovery of better ASO carriers, we developed a “switch-on” reporter system based on antisense-mediated toehold-switch activation (Fig. 1). We show that this system reports ASO uptake in *E. coli* and *Salmonella* and that it can be used to investigate mechanisms of CPP-PNA transport. The switch-on assay presents a simple and semi-quantitative method that lends itself as a building block for future high-throughput screens. Additionally, our work demonstrates that toehold switches can be activated by biostable RNA mimics. This opens their potential application in synthetic biology as tools for orthogonal regulation of multiple genes, each under the control of a different RNA toehold switch, for example, for the design of synthetic gene expression networks.

### The switch-on reporter is a versatile tool for high-throughput screens for ASO carriers

ASO uptake into bacteria can be studied using fluorophore labeling of CPP-PNAs (44), mass spectrometry (22, 27), measurement of the antibacterial activity (MIC) of PNAs targeting essential genes (12), or analysis of the transcriptomic responses by using RNA-seq (13, 22). Our assay is a simple alternative that requires no chemical modification of the CPP-PNA (unlike fluorophore labeling) and minimal sample preparation (compared to mass spectrometry and RNA-seq). We validated our reporter assay using two different approaches. First, we showed reduced activation of the toehold switch by KFF-PNA_toe in a bacterial strain lacking the inner membrane transporter SbmA, which is essential for the transport of KFF-PNA conjugates into bacteria (27). Second, we found a good overall correlation of CPP-PNA_toe-mediated increase in sfGFP fluorescence with traditional MIC assays if the identical CPPs were fused to a PNA targeting the essential gene *acpP*. All CPPs that mediated reporter activation showed antibacterial activity, with only one exception (Seq118 in *E. coli* BW25113). This indicates a low probability of predicting false positives if the reporter assay is used to screen for CPPs for antisense antibiotic delivery. We also observed that L5a was antibacterial although it did not activate the reporter in *Salmonella*, suggesting that other factors beyond ASO delivery can contribute to the antibacterial activity of a CPP-PNA conjugate.

The dynamic range and sensitivity of the assay in the *E. coli* K12 strains are lower than in *Salmonella* and UPEC 536 strains. The MIC assays also show a lower sensitivity of the K12 strains to CPP-*acpP* (Fig. S5). This might be due to differences in the composition of the bacterial envelope: *E. coli* K12 strains express rough lipopolysaccharide (LPS), while the *Salmonella* and UPEC 536 strains have smooth LPS (45). This hypothesis is supported by previous reports demonstrating that LPS composition affects the susceptibility of bacteria to KFF-PNA conjugates (46). To what extent envelope variations or other unknown factors contribute to the sensitivity of bacterial strains to the reporter assay will need further investigation.

We think that the switch-on reporter assay described here can be developed into a high-throughput screening platform for the discovery of ASO carriers. Bacteria can be treated with a single concentration of each CPP conjugated to PNA_toe (or an optimized version thereof) unlike in MIC determination experiments, which require serial dilutions of the ASO constructs. The fluorescence output can be quantified easily to rank and select CPP based on their efficiency of ASO delivery. The plate reader offers a simple initial read-out but is limited in sensitivity when the fluorescence signal is low. Flow cytometry requires more sample preparation but offers higher sensitivity, which might be important to achieve the dynamic range needed to screen some bacterial strains like the *E. coli* K12 strains used here (Fig. 10).

### The toehold switch reporter reveals different dynamics of CPP-mediated PNA delivery

Our screen of 10 CPPs revealed different patterns of upregulation in flow cytometry. For example, we observed a rapid but transient increase in fluorescence for some CPPs (e.g., KFF) and a sustained increase for others (e.g., RXR). This variation can likely be explained by the chemistry and stability of these carriers. The KFF peptide is sensitive to proteases and degraded in nutrient-rich media such as MHB (27). By contrast, RXR is composed of a non-canonical 6-aminohexanoic acid and is resistant to proteolytic degradation (47). Therefore, the different dynamics in fluorescence increase could inform about the CPP stability in bacterial culture. Another observed difference among CPPs was that some (KFF, RXR, and ANT) induced strong and rapid upregulation within 4 h, while others, like TAT, were slower, with robust reporter activation only after prolonged incubation. Previously, we have shown that KFF- and RXR-*acpP* eradicate bacteria after 15 min, while TAT achieves the same effect in 40–60 min (13). This indicates that the upregulation of fluorescence from the toehold switch reporter resembles the dynamics of delivery albeit at longer time scales.

### Limitations and optimization of the assay

We established the switch-on reporter assay using a well-studied strain of *Salmonella* as the model and validated its applicability in other enteric bacteria. In this study, we have tested two PNAs that target different regions of the stem-loop. PNA_toe was more efficient than PNA_mid, which is consistent with previous reports identifying the toehold as the most crucial site for stem displacement (48). When the activating RNA bound in proximity to the RBS (as does PNA_mid), switch activation was lower (33). This might be caused by interference with ribosome binding, as shown for bacterial sRNAs that repressed translation of target mRNAs by base-pairing just outside the RBS (49, 50).

Our assay shows a high dynamic range in *Salmonella* and UPEC 536, but the range is more limited in *E. coli* K12 strains. Strategies to enhance the sensitivity and dynamic range of the assay could involve the optimization of the switch and the activator ASO sequence. First, mutations in the stem of the toehold switch will likely increase sensitivity, although these mutations, which partly open the stem-loop, bear the risk of higher basal fluorescence. While this might compromise the signal-to-noise ratio, a systematic screen of the TS7 sequence with single or double mutations might nevertheless produce TS7 sequences optimized for application to short ASOs. Such an approach has already been established for the study of 5′ mRNA regions and their regulation by bacterial sRNAs (51). Additionally, as reported by Green et al., the length of the loop domain can be increased to enhance the protein output and the dynamic range of the assay (33). Second, the activator could be improved. Our lead PNA (PNA_toe) achieves up to 60-fold activation of the TS7 toehold switch, which is substantially lower than the up to 300-fold activation achieved by the 30mer sRNA in the original work (33). Since strand displacement-assisted activation depends on the length of the competing oligomer (48), longer PNAs might be able to achieve higher activation. However, longer PNAs are usually not internalized efficiently and, therefore, may not necessarily lead to higher activation *in vivo* (12, 44).

The dynamic range of the assay will also depend on the binding affinity between the ASO modality and RNA. ASO backbones such as PMOs have lower binding affinity to RNA compared to PNAs. Therefore, longer oligomers may be required to activate switches like TS7 similar to activation with antisense RNAs (33). The use of other ASO backbones to activate the toehold-switch assay will require systematic optimization. To identify an optimal activator, one could tile the 5′ flank of the TS7 toehold sequence with ASOs of varying lengths. For example, a recently developed high-throughput synthesis approach can be used to synthesize hundreds of PNA conjugates and to test them *in vivo* (G. Danti, L. Popella, J. Vogel, and H. Maric, personal communication). It is important to consider factors beyond complementarity such as an ASO’s melting temperature (*T*_m_), percentage of purines, self-complementarity and off-target binding, all of which might affect the activity of specific ASO sequences (12). To further improve the assay, independent expression of a different fluorescence protein from the same plasmid could be used to assess potential copy number variations of the reporter plasmid and toxicity of the CPP-ASO. There is a large repertoire of fluorescent proteins that lend themselves as reporters in bacteria, and many of these can be read out in parallel albeit at different wavelengths (52).

While we envision that our work will serve as a template for developing switch-on reporter assays for other pathogens of interest, including Gram-positive bacteria such as *Staphylococcus aureus*, *Enterobacter faecium,* and *Clostridium difficile*, our data show that reporter activation varies between bacterial species, growth media, ASO concentration and design, reporter protein, and the time of analysis post-ASO treatment. Consequently, implementing this assay as a screening platform in other bacteria will require optimization of these factors. For example, some reporter proteins such as GFP do not fold under anaerobic conditions necessary for the growth of some bacterial species, e.g., *C. difficile*; mCherry might be an alternative here. Recent work in *Fusobacterium nucleatum* has established conditions for the use of different fluorescent proteins under oxygen tension (53), which might help to set up RNA toehold switch assays in a range of other anaerobic bacteria.

### Outlook

Due to the modular nature of CPP-ASOs, precision manipulation of bacterial communities can be achieved either by targeting a species-specific RNA sequence or by using carriers that facilitate selective transport into only one specific species in the community. The assay developed here will enable the high-throughput discovery of novel carriers for the delivery of antisense PNAs, by informing about (i) PNA sequence-independent confounding effects; (ii) uptake kinetics and mechanisms; as well as (iii) species-selectivity of uptake. While we have used our assay to screen CPPs, it may be extended to other classes of carriers, such as nanoparticles (54–57) and siderophores (58, 59). Identifying alternative ASO carriers would allow the use of other ASO backbones such as the negatively charged 2′-4′ carbon bridged-locked nucleic acids (LNA) and 2ʹ-O-methoxyethyl (2ʹ-MOE) which have so far been difficult to deliver with CPPs (22). This could dramatically expand the chemical space for ASOs targeting microbes.

## MATERIALS AND METHODS

### Media, bacterial strains, PNAs, and peptide-conjugated PNAs (CPP-PNAs)

The following strains were used in this study: *Salmonella enterica* serovar Typhimurium strain SL1344 (provided by D. Bumann, MPIIB Berlin, Germany; internal strain number JVS-1574; NCBI GenBank: FQ312003.1), uropathogenic *E. coli* 536 (UPEC 536, internal strain number JVS-12054; NCBI GenBank: CP000247.1), *E. coli* BW25113 (internal strain number JVS-12547; NCBI GenBank: CP009273.1), and *E. coli* MG1655 (internal strain number JVS-5709; NCBI GenBank: U00096.3). All bacterial strains are listed in Table S5.

M9 medium (1 L, 5×) was prepared with 64 g Na_2_HPO_4_·7H_2_O, 15 g KH_2_PO_4_, 2.5 g NaCl, 5 g NH_4_Cl, 2 mM MgSO_4_, and 1× M9 medium was obtained by diluting 5× M9 in water supplemented with 0.1 mM CaCl_2_, 0.4% glucose, 0.2% CAS, 0.004% histidine (0.8%−2.5 mL), 25 µL 10 mg/mL thiamine. Luria-Bertani broth (LB) was made from 10 g tryptone (Carl Roth), 5 g yeast extract, and 0.5 g NaCl. Mueller Hinton broth (MHB) was purchased from BD Difco, Thermo Fisher Scientific. Strains were streaked on LB plates and incubated overnight at 37°C. Liquid cultures were inoculated in MHB, LB, or M9 minimal medium under agitation at 37°C. Antibiotics were added if needed at the following final concentrations: 100 µg/mL ampicillin, 50 µg/mL kanamycin, 20 µg/mL chloramphenicol.

PNAs and peptide-conjugated PNAs (CPP-PNAs) were purchased from Peps4LS GmbH (Heidelberg, Germany). The quality and purity of these constructs was verified by mass spectrometry and HPLC (Peps4LS GmbH). CPP-PNA were synthesized together on the same resin and do not have a linker between the CPP and the PNA. Unconjugated PNAs and CPP-PNAs (Table S6) were dissolved in water and heated at 55°C for 5 min before usage. The concentration of the constructs was determined using a NanoDrop spectrophotometer (*A*_260_). Aliquots of PNAs, CPP-PNAs, and peptides were stored at –20°C and heated at 55°C for 5 min before preparing the respective working dilutions. Low-binding pipette tips and Eppendorf tubes (Sarstedt) were used for handling PNAs.

### *In vitro* transcription

Templates for T7 RNA polymerase-mediated *in vitro* transcription were generated via overlap polymerase chain reaction (PCR) to generate GFP fusion constructs. For this, oligonucleotides were designed to amplify TS7 from an Addgene plasmid (plasmid #107355), and *gfp* from pXG10 plasmid backbone (35) using a protocol from Popella et al. (12). The amplicon of TS7 spanned nucleotides –41 to +30 relative to the translational start codon as described by Green et al. (33). The PCR for the overlap fusion was performed by using four oligonucleotides: (i) forward primer (JVO-20850) annealing at the 5′ end of TS7 (A T7 promoter sequence was attached to the 5′ end to allow subsequent transcription of the TS7::*gfp* fusion construct *in vitro*), (ii) a reverse primer (JVO-21361) amplifying TS7 including a 30 nucleotide *gfp* overlap at the 3′ end (to enable fusion to *gfp*, (iii) forward primer annealing at the 5′ end of *gfp* coding sequence (JVO-19762), and (iv) reverse primer binding the 3′ end of *gfp* (JVO-19763). All oligonucleotides were purchased from Eurofins Genomics and sequences are listed in Table S3. For PCR amplification, 10 ng of each plasmid was used as a template, and all four oligonucleotides were added at a molar ratio of 10:1:1:10 according to references (60). The PCR program was set as follows: 1 min, 98°C; 40× cycles of 98°C for 10 s, 60°C for 20 s, 72°C for 40 s; 72°C for 10 min, then stored at 4–8°C until further purification. The PCR product was purified using agarose gel electrophoresis followed by PCR Clean-up (Macherey-Nagel) according to the manufacturer’s instructions, and DNA concentration was quantified by using NanoDrop spectrophotometer (*A*_260_).

After agarose gel-based purification, approximately 500 ng of template DNA was subjected to 20 µL *in vitro* transcription reactions following the manufacturer’s instructions (MEGAscript T7 kit, Ambion/Thermo Scientific). The samples were incubated at 37°C for 4 h, after which 2 units (U) of Turbo DNase were added to each reaction for an additional 15 min. One hundred fifteen microliters of water, 15 µL ammonium acetate stop solution, and 3× volume of absolute ethanol were added sequentially to precipitate RNA at –80°C overnight. The samples were then centrifuged, and pellets were washed with 70% ethanol and finally resuspended in 20 µL ultrapure water. RNA concentration was measured with Qubit (Fisher Scientific). RNA gels (6% polyacrylamide, 7 M urea) were prepared and stained with StainsAll (Sigma-Aldrich) to verify the expected product size and RNA integrity.

### *In vitro* translation and western blotting

To assess PNA-mediated activation of reporter translation *in vitro*, the PURExpress *In Vitro* Protein Synthesis Kit (New England Biolabs, E6800L) was used according to the manufacturer’s instructions. *In vitro* transcribed RNA was prepared as described above and used in all translation reactions to ensure a precise 100 nM final RNA concentration in each sample. Before starting the translation reaction, *TS7::gfp* RNA was heat-denatured (95°C for 1 min), chilled on ice, and incubated at 37°C for 5 min in the absence or presence of PNA_toe, PNA_ctrl or equal volume of water as negative control. PNA:RNA titrations corresponding to 10:1, 5:1, 2:1, and 1:1 molar ratios were tested. One microliter of 1 µM RNA was translated in reactions containing 1 µL of 10 µM, 5 µM, 2 µM, or 1 µM PNA solutions. Next, PURExpress *in vitro* translation solutions A and B were added and handled as described. Each *in vitro* translation reaction was performed at a final volume of 10 µL, containing 4 µL solution A, 3 µL solution B, and 1 pmol of *in vitro* transcribed RNA (adjusted to a final concentration of 100 nM). The reactions were incubated for 2 h at 37°C after which the tubes were immediately placed on ice. The protein samples were mixed with reducing protein loading buffer (final 1×: 62.6 mM Tris-HCl [pH 6.8], 2% SDS, 0.1 mg/mL bromophenol blue, 15.4 mg/mL DTT, 10% glycerol) and denatured at 95°C for 5 min. The samples were separated on an SDS-PAA (12% PAA) gel followed by subsequent semi-dry western blot transfer on nitrocellulose membranes to visualize translated protein amounts. The membranes were stained with Ponceau S (Sigma-Aldrich) to check for equal loading. Afterward, membranes were washed with 1× TBST (Tris-buffered saline with 10% Tween), blocked in 5% skim milk (in 1× TBST), and probed with anti-GFP antibody (1:1,000; Sigma-Aldrich) in 3% bovine serum albumin (BSA) solution made in 1× TBST overnight. After this, the membrane was washed and incubated with an HRP-conjugated secondary antibody (1:10,000; α-mouse, ThermoScientific) in 3% bovine serum albumin (BSA) solution made in 1× TBST. Protein levels were detected using an ImageQuant LAS 500 (GE Healthcare Life Sciences) following incubation with a self-made developing solution (0.3 µL 30% H_2_O_2_, 2 mL Solution A (20 mL 1 M Tris-HCl [pH 8.6], 180 mL autoclaved milliQ H_2_O, and 50 mg luminol sodium salt [Sigma A4685])], 200 µL solution B (55 mg para-hydroxycoumaric acid [Sigma C9008] dissolved in 50 mL DMSO]). Images were processed, and band intensities were quantified using ImageJ.

### Cloning of translational TS7-reporter plasmids

All plasmids used as PCR or cloning templates or that were generated within this study are listed in Table S4. pNP1 sfGFP toehold switch 7 was a gift from James Collins (Addgene plasmid # 107355; https://www.addgene.org/107355/; RRID:Addgene_107355). DNA oligonucleotides used for amplification and sequencing were purchased from Eurofins Genomics and are listed in Table S3. Oligonucleotide primers were designed to include the NsiI (for forward primer, JVO-21317) and NheI (for reverse primer, JVO-21318) to amplify TS7 (–41 to +30 relative to the translational start codon) from the Addgene plasmid (Plasmid #107355). The TS7 insert was generated using the PCR program as follows: 1 min, 98°C; 40× cycles of 98°C for 10 s, 58°C for 20 s, 72°C for 40 s; 72°C for 10 min, then stored at 4–8°C until further purification. The PCR product of the correct size (118 bp) was purified with 1% agarose gel electrophoresis followed by PCR Clean-up (Macherey-Nagel) according to the manufacturer’s instructions. The pXG10-SF backbone (35) as well as the TS7 insert were cut with NsiI/NheI for subsequent cloning. For the *TS7::mCherry* construct, *mCherry* was PCR amplified (JVO-19329/19330) and cloned into TS7 containing pXG10-SF (pPS002 backbone), by restriction cloning at NheI/XbaI sites. For the *TS7::mNeonGreen* construct, fusion PCR was used to generate the inserts (JVO-21317/21512/JVO-21511/20632) including NheI and XbaI restriction sites. The vector pXG10-SF (35) was cut at NsiI/XbaI and *TS7::mNeonGreen* was inserted using restriction cloning. Single-nucleotide mutagenesis for the construction of mutated toehold switches, TS7-Mut_9, TS7-Mut_13, and TS7-Mut_18 were performed on pPS002 using primer pairs specified in Table S4. Briefly, mutations were generated using the PCR program as follows: 30 s, 98°C; 40× cycles of 98°C for 10 s, 52°C for 30 s, 72°C for 2 min; 72°C for 10 min, then stored at 4–8°C until further purification. The plasmid was purified with 1% agarose gel electrophoresis followed by PCR Clean-up (Macherey-Nagel) according to the manufacturer’s instructions. Plasmids were sequenced and validated using primers listed in Table S1. *E. coli* TOP10 (Invitrogen) was used for all cloning procedures.

### CPP-PNA treatment of bacteria for sfGFP measurement

Overnight cultures of respective bacterial strains (listed in Table S5) were diluted 1:100 in fresh MHB and grown to OD_600_ 0.3. Overnight and secondary cultures were prepared with or without chloramphenicol (20 µg/mL) depending on the antibiotic resistance profile of the strain. Ninty-five microliters of the 0.3 OD_600_ bacterial suspension was distributed in the 96-well plate and mixed with 5 µL water or 20× CPP PNA working solutions (150 µM, 100 µM or 50 µM stocks in water). An equivalent volume of water was added to the bacteria to serve as untreated control. For testing the activity in other growth media (M9 minimal medium and LB), the bacteria were cultured in the respective media and treated as described above.

The PNA-treated samples were incubated in the plate reader for 4–24 h at 37°C, with continuous double-orbital shaking (237 counts per minute). The fluorescence (λ_ex_ 480 nm/λ_em_ 510 nm) and OD_600_ were recorded in a Synergy H1 plate reader (Biotek) by measuring every 20 min. The bacteria were sampled at various time points post-treatment and further analyzed by flow cytometry.

Normalization of fluorescence intensity (FI_norm_) = $\frac{FI\;\left(sample\right)-FI\;\left(medium\;background\right)}{OD}$

Fold upregulation in fluorescence intensity (FI) from the plate reader was calculated as follows:

fold upregulation = $\frac{FI\;\left(normalized\;sample\right)}{FI\;\left(normalized\;control\right)}$

### Flow cytometric measurement and data analysis

Bacterial cells were treated as described above, fixed with 4% paraformaldehyde (PFA) in phosphate-buffered saline (PBS) after the indicated time points, and stained with 1 µg/mL DAPI for flow cytometric measurement of DAPI-positive bacterial cells. Cell suspensions were diluted by a factor of ~100–1,000 in PBS and sampled from 96-well plates to achieve ~2,000 counts/s for flow cytometric measurements. Flow cytometry was performed using the Agilent Novocyte flow cytometer with a high-throughput sampler, and 100,000 events were recorded per sample. The DAPI fluorescence was detected using the Pacific Blue channel; sfGFP and mNeon were detected in the FITC channel, and mCherry fluorescence in the PE-Texas Red channel. The lower threshold for FSC-H was set to 2,000 to eliminate the potential artifacts from smaller particles such as dust, bubbles, or cell debris. The wild-type strains without plasmids served as a control to detect the background fluorescence, termed bacterial background. A contour plot with FSC and SSC, each with bins defined on a logarithmic scale was used to select singlets. The gating strategy is described in Fig.S6. The median fluorescence intensity was used for calculating the fold upregulation of fluorescence. Flow cytometry data sets were analyzed using the Novocyte software (Agilent).

Fold upregulation in median fluorescence intensity (FI) from flow cytometry was calculated as follows:

fold upregulation = $\frac{FI\;\left(sample\right)-FI\left(bacterial\;background\right)}{FI\;\left(water\;control\right)-FI\left(bacterial\;background\right)}$



The fold upregulation was calculated from at least two independent experiments.

### Western blot analysis of sfGFP protein levels post ASO treatment

Overnight cultures of *Salmonella* and *E. coli* BW25113 strains with the *TS7::sfgfp* expressing plasmid (listed in Table S5) were diluted 1:100 in fresh MHB and grown to OD_600_ 0.3. Overnight and secondary cultures were prepared with chloramphenicol (20 µg/mL). Ninety-five microliters of the 0.3 OD_600_ bacterial suspension was distributed in the 96-well plate and mixed with 5 µL water or 20 × CPP PNA working solutions (100 µM stocks in water). An equivalent volume of water was added to the bacteria to serve as untreated control. The PNA-treated samples were incubated in the plate reader for 4 h at 37°C, with continuous double-orbital shaking (237 cpm). The cells were collected after centrifugation at 4°C and 10,000 rpm for 10 min, washing once in 1× PBS and resuspended in 1× protein loading buffer (62.6 mM Tris-HCl [pH 6.8], 2% SDS, 0.1 mg/mL bromophenol blue, 15.4 mg/mL DTT, 10% glycerol). The cells were denatured at 95°C for 5 min and stored at −20°C for WB analysis.

To detect sfGFP protein levels, samples were separated on a 12% SDS-PAA gel and transferred onto a nitrocellulose membrane via semi-dry western blotting. The membrane was probed with an α-GFP antibody (Sigma; 1:1,000 in 1× TBST containing 3% BSA) at 4°C overnight. After washing in 1 × TBST, the membrane was incubated with a secondary HRP-conjugated anti-mouse antibody (Thermo Scientific; 1:5,000 in 1× TBST containing 1% BSA) at RT for 1 h. Excess antibody was removed by washing in 1× TBST, and the membrane was developed with Amersham ECL Select Western Blotting Detection Reagent Kit (Cytiva) adding 0.3 µL 30% H_2_O_2_.

GroEL was used as a loading control. The membrane was stripped, dried in air, and washed with TBST buffer followed by blocking with 10% skim milk in 1× TBST for 1 h. This was followed by incubation overnight with α-GroEl (Sigma; 1:10,000 in 3% BSA in 1× TBST) at 4°C overnight. After washing in 1 × TBST, the membrane was incubated with a secondary HRP-conjugated antibody (α-rabbit, Thermo Scientific; 1:10,000 in 1× TBST containing 1% BSA) at RT for 1 h and washed in 1× TBST. The membrane was developed by Amersham ECL Select Western Blotting Detection Reagent Kit (Cytiva) adding 0.3 µL 30% H_2_O_2_.

### Minimal inhibitory concentration assay

The broth microdilution method was applied for the determination of MIC values according to a previously modified protocol based on the Clinical and Laboratory Standards Institute guidelines (36, 61). Overnight bacterial cultures were diluted 100-fold in fresh MHB and grown to an OD_600_ 0.5. The obtained culture was diluted in fresh MHB to adjust a final cell concentration of ∼10^5^ cfu/mL. Ninety-five microliters of the diluted bacterial solution was dispensed into a 96-well plate (Thermo Fisher Scientific). Five microliters of 20 × CPP-PNA working solutions (ranging from 400 to 6.25 µM) or an equivalent volume of water as a negative control was added (CPP-PNAs included in Table S6). Growth (OD_600_) was monitored in a Synergy H1 plate reader (Biotek) by measuring every 20 min with continuous double-orbital shaking (237 cpm) at 37°C for 24 h. The MIC was determined as the lowest concentration of CPP-PNA, which inhibited visible growth in the wells (OD_600_ < 0.05).

## SUPPLEMENTAL MATERIAL

Supplemental material mbio.03983-24-s0001.pdf Figures S1-S6; Tables S1-S6.

ASM does not own the copyrights to Supplemental Material that may be linked to, or accessed through, an article. The authors have granted ASM a non-exclusive, world-wide license to publish the Supplemental Material files. Please contact the corresponding author directly for reuse.
