# Supplementary material for: RNA toehold switch-based reporter assay to assess bacterial uptake of antisense oligomers
Source: mBio. 2025 Mar 4;16(4):e03983-24. doi: 10.1128/mbio.03983-24 (PMC12017328; doi:10.1128/mbio.03983-24)
Supplement: Supplemental material — Figures S1-S6; Tables S1-S6. [file mbio.03983-24-s0001.pdf]

Supplementary information

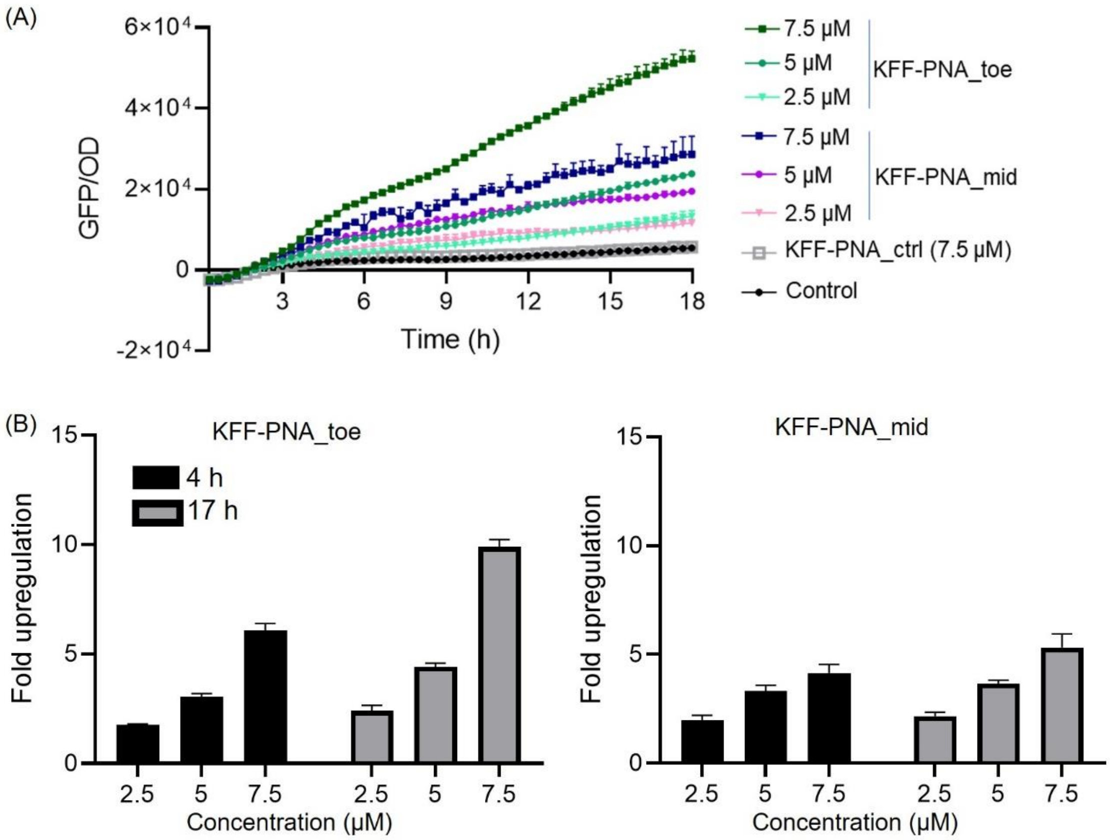

Figure S1. Assessment of fluorescence induction in *Salmonella* (transformed with a plasmid expressing *TS7::sfGFP*) post-treatment with KFF-PNAs targeting different regions of the toehold switch. (A) Normalized fluorescence intensity over time and (B) fold-upregulation of fluorescence of bacteria treated with KFF-PNA_toe and PNA_mid, respectively, measured in a microplate reader. Bacteria were cultured to an OD_{600} of 0.3 and treated with 2.5, 5, and 7.5 µM concentrations of each KFF-PNA. A non-targeting KFF-PNA (KFF-PNA_ctrl) was tested to assess non-specific upregulation from KFF-PNA at the highest concentration (7.5 µM). 'Control' denotes treatment with an equal volume of water. The fluorescence intensity obtained from the microplate reader was normalized relative to the OD_{600} to account for the decrease in fluorescence due to growth retardation. Bars indicate fold upregulation of PNA-treated samples relative to the water control and error bars indicate the standard deviation calculated from two independent experiments.

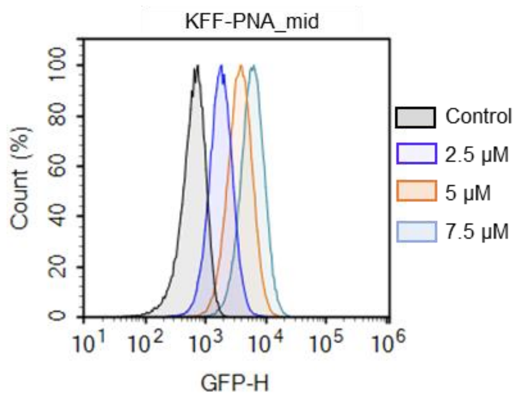

Figure S2. Flow cytometric analysis of activation of *TS7::sfGFP* with KFF-PNA_mid. The flow cytometry histogram represents mean fluorescence intensity of sfGFP 4 h post-treatment of *Salmonella*. *Salmonella* were cultured to an OD_{600} of 0.3 and treated with 2.5, 5, and 7.5 µM concentrations of KFF-PNA_mid. The water-treated sample served as the control condition (Control).

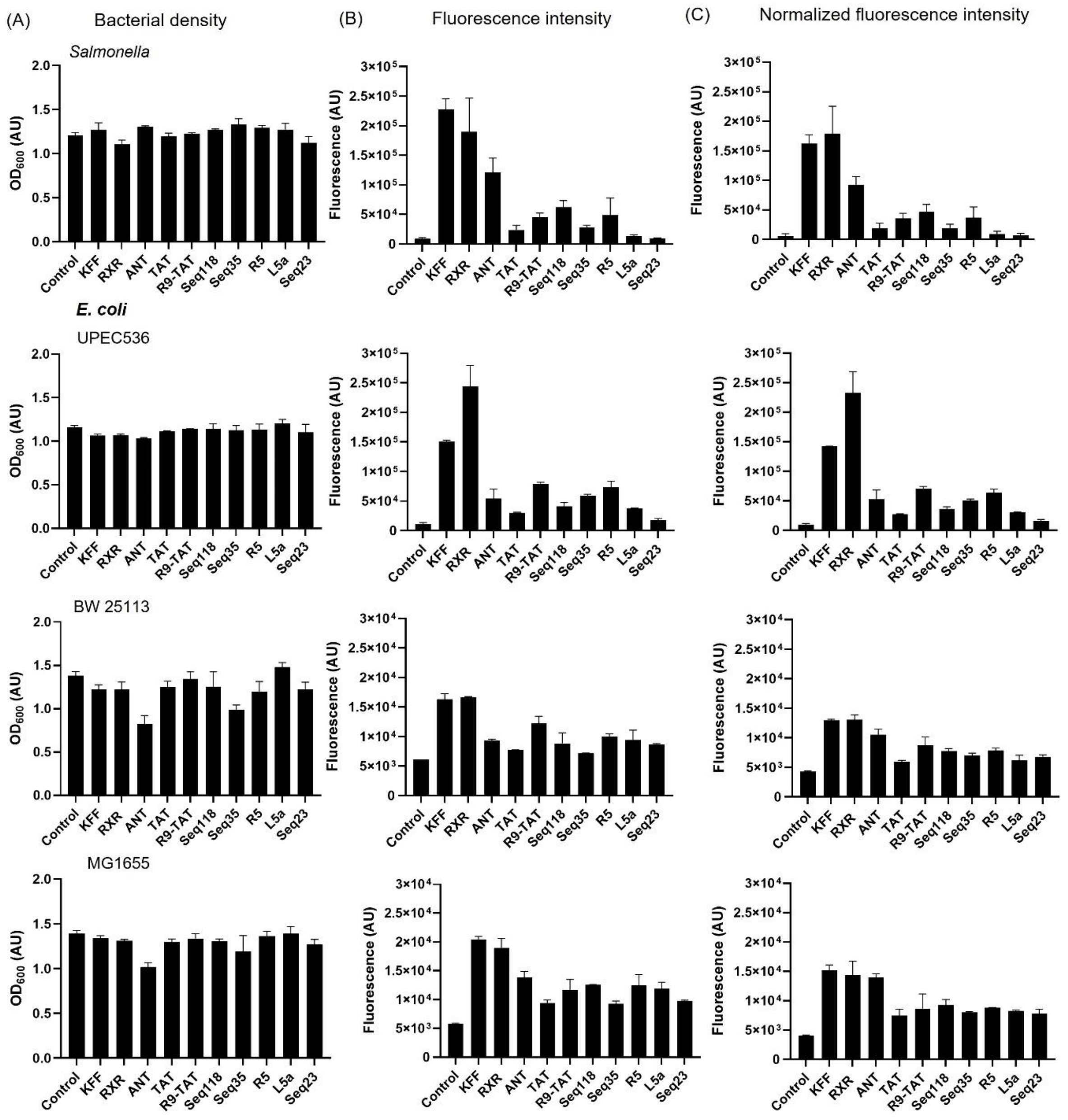

Figure S3. Fluorescence intensity is normalized to OD_{600} to enable comparison of PNA delivery efficacy. (A) OD_{600}, (B) raw fluorescence intensity and (C) fluorescence intensity normalized to OD_{600} of bacteria expressing *TS7-Mut_9::sfgfp* constructs, 17 h post-treatment with 5 µM concentrations of CPP-PNA_toe measured in a microplate reader. Bacteria were cultured to an OD_{600} of 0.3 and treated with CPP-PNA. The water-treated sample served as control. The fluorescence intensity obtained from the microplate reader was normalized relative to the OD_{600} to account for the decrease in fluorescence due to growth retardation. Bars indicate the mean fluorescence intensity of PNA-treated and control samples. Error bars indicate the standard deviation of mean fold upregulation calculated from two independent experiments.

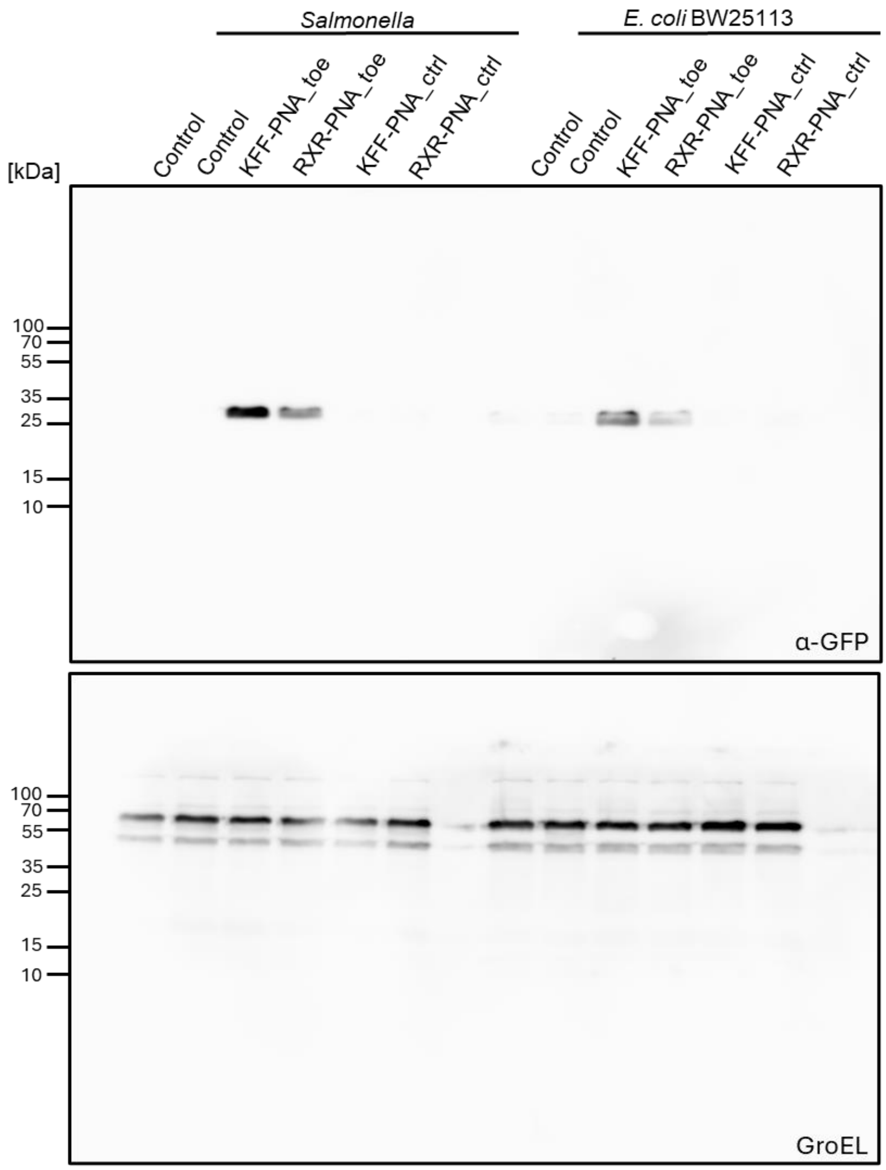

Figure S4. Western blot analysis of antisense-mediated activation of toehold switch-controlled sfGFP expression in *Salmonella* and *E. coli*. Bacteria expressing *TS7-Mut_9::sfgfp* constructs were treated with 5 µM concentrations of KFF- or RXR-PNA_toe for 4 h. As negative controls, we included an equal volume of water-treated samples (Control) and the corresponding CPP-conjugates of a non-targeting PNA_ctrl (KFF-PNA_ctrl, RXR-PNA_ctrl). Western blot-based detection of sfGFP was performed using a monoclonal α-GFP antibody (upper panel). GroEL was used to control for equal loading (lower panel).

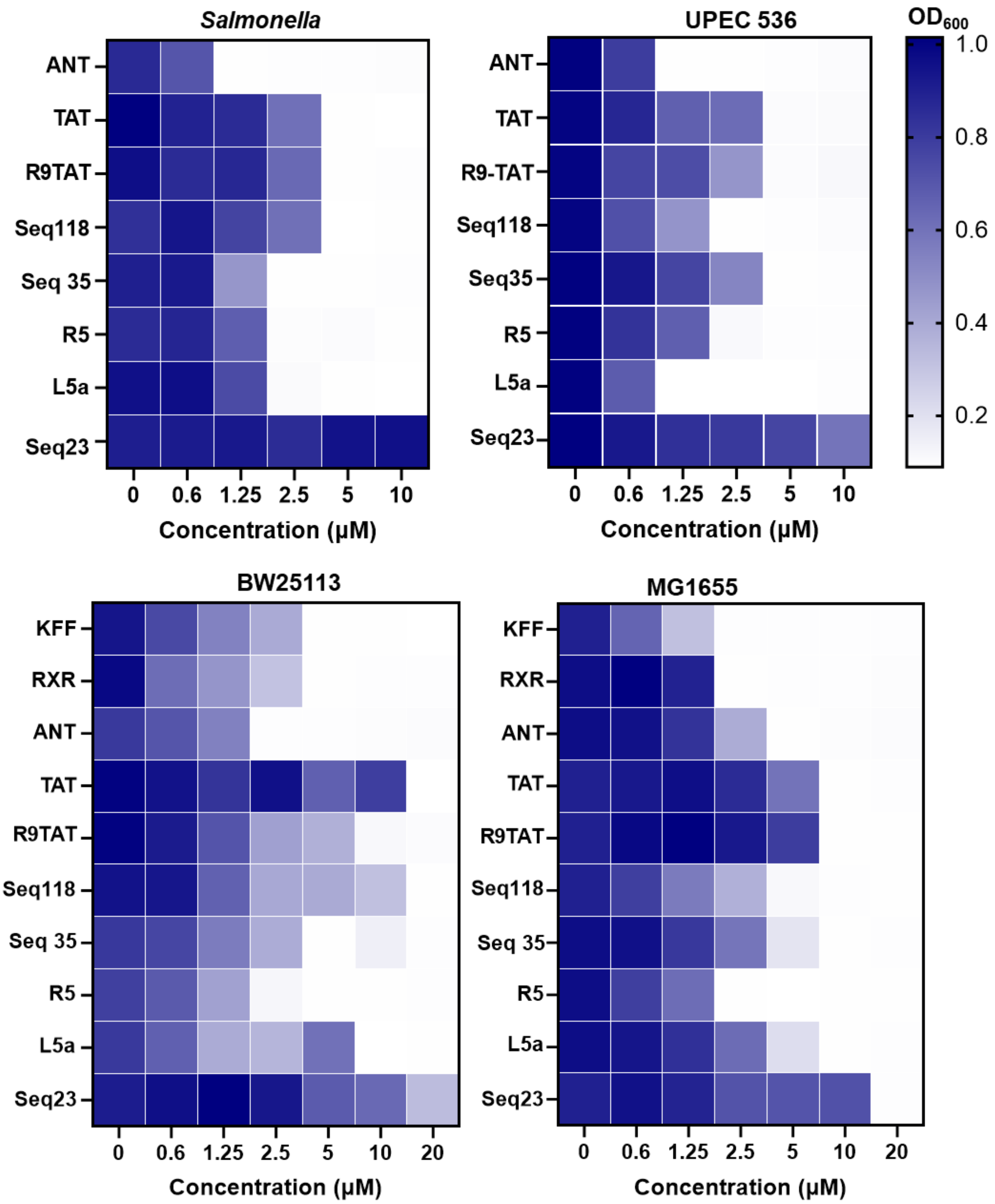

Figure S5. MIC determination validates the reliability of the switch-on reporter assay. The antibacterial activity of the 10 CPPs when conjugated to an anti-*acpP* PNA was tested against various *wt* strains of *Salmonella* and *E. coli.* The heatmaps indicate the OD_{600} of bacteria 20 h post-treatment with varying concentrations of CPP-PNAs. Row titles indicate CPP; all CPPs were conjugated to the PNA sequence CTCATACTCT, antisense to the translation initiation region of the *acpP* mRNA. Column titles indicate the concentration of CPP-*acpP*.

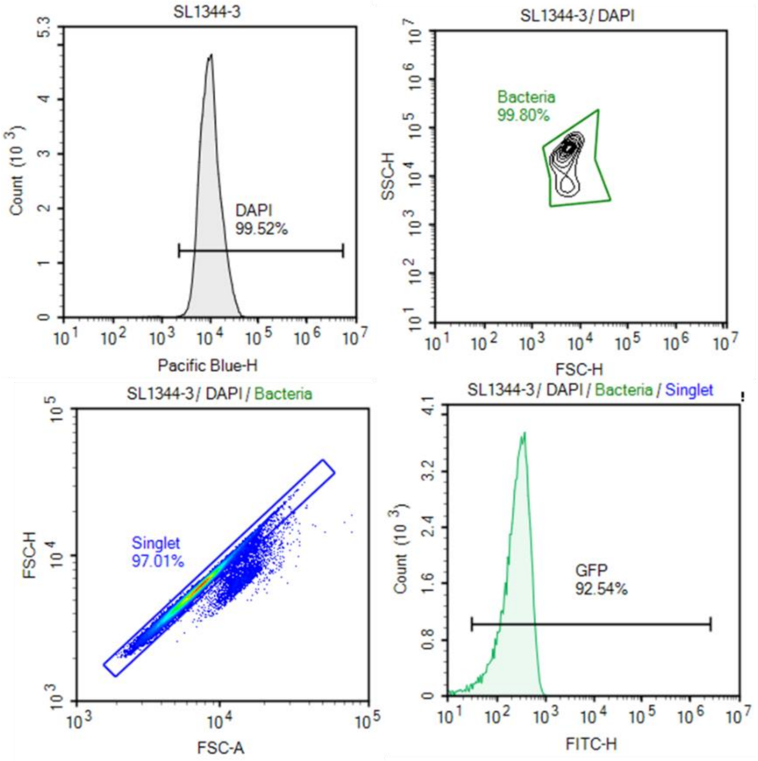

Figure S6. Gating strategy for flow cytometry. *Salmonella* were fixed and stained with DAPI to include only intact cells and 100,000 events were recorded in the Pacific Blue channel. The fluorescence of DAPI was recorded using the Pacific Blue channel; sfGFP was detected in the FITC channel. The FSC-H threshold was set to 2000 to avoid artifacts from debris, bubbles or dust.

Table S1. Fluorescence intensity of *Salmonella* carrying plasmids encoding *TS7-Mut_9::sfgfp*, 17 h post-treatment with a non-targeting PNA sequence (PNA_ctrl) conjugated with various CPPs measured using a microplate reader

CPP	PNA	Fluorescence Intensity	Fold upregulation
ANT	pna2	12322	1.2
RXR	pna19	12476	1.2
KFF	pna22	10120	0.9
TAT	pna97	13563	1.3
Seq118	pna183	12112	1.1
Seq35	pna184	12650	1.2
R9-TAT	pna186	11993	1.1
L5a	pna1157	9284	0.9
-	Water control*	10667	1

Internal PNA numbers are given in column 2.
Fold upregulation was calculated relative to the water-treated control
*Water control denotes the bacterial sample treated with only an equivalent volume of water, serving as the baseline.

Table S2. Median fluorescence intensity of *Salmonella* and *E. coli* expressing *TS7-Mut_9::sfGFP*, at various time points post-treatment with non-specific PNA, PNA_ctrl sequence conjugated with various CPPs measured using flow cytometry

**Sample**	**Median Fluorescence (GFP-H)**			
	Salmonella	*E. coli* MG1655	*E. coli* BW25113	UPEC 536
**4 h**				
ANT	523	554	564	650
RXR	488	537	585	466
KFF	491	549	577	634
TAT	537	549	582	643
Seq118	526	553	585	635
Seq35	509	549	596	639
R9-TAT	476	570	610	646
L5a	470	553	602	643
Water control*	479	554	601	634
**6 h**				
ANT	564	481	519	662
RXR	445	471	490	420
KFF	507	482	498	642
TAT	595	476	499	658
Seq118	537	477	501	642
Seq35	527	479	510	653
R9-TAT	531	423	515	646
L5a	542	487	499	650
Water control*	500	488	503	644
**17 h**				
ANT	748	548	488	926
RXR	472	540	494	596
KFF	669	542	458	835
TAT	716	556	444	896
Seq118	594	545	440	1099
Seq35	641	567	476	993
R9-TAT	530	560	455	911
L5a	661	554	443	953
Water control*	656	548	447	963

*Water control denotes the bacterial sample treated with only an equivalent volume of water, serving as the baseline.

Table S3. List of oligonucleotides used in this study

Name	Sequence	Comment
JVO-16358	AGCAGCCGTAATAAAGATAAGAAGTTAGCAGGAGAGCATG GTGTAGGCTGGAGCTGCTTC	Fw primer for deleting sbmA in *S. typhimurim* based on pkD4
JVO-16359	TTAGCTAAAAGAGTGGGTCACTTCCTGAACTGGCTGACCAGGTCCATATGA ATATCCTCCTTAG	Rv primer for deleting sbmA in *S. typhimurim* based on pkD4
JVO-16360	GCAGAAATGCGAACTAAGCG	Fw primer for SbmA in *S. typhimurim* mutant verification
JVO-16361	GCCAGCCAATATGGCAACCG	Rv primer for SbmA in *S. typhimurim* mutant verification
JVO-19329	GGCTGCTAGCATGGTGAGCAAGGGCGAGGA	Fw primer annealing to mCherry; contains NheI site
JVO-19330	GCGTCTAGACTACTTGTACAGCTCGTCCATG	Rv primer annealing to mCherry containing XbaI
JVO-19762	GCTAGCAAAGGAGAAGAACTTTTCAC	Fw oligo to amplify GFP from pXG10
JVO-19763	TTATTTGTAGAGCTCATCCATGCC	Rv oligo to amplify GFP from pXG10
JVO-20598	AAGTGTTGGCCATGGAACAGG	Sequencing primer annealing to sfGFP
JVO-20632	GCGTCTAGATTATTTATACAGTTCATCCATACCC	Rv oligo priming in gene encoding mNeonGreen; contains XbaI site
JVO-20850	GTTTTTTTTAATACGACTCACTATAGGCCCGCGAAATTAATACGACTCAC	Fw with T7 promoter sequence to synthesize TS7 for in vitro transcription
JVO-21317	GTTTTTATGCATCCCGCGAAATTAATACGACTCAC	Fw primer for TS7 containing NsiI site
JVO-21318	GGCTGCTAGCCATCTTTTGCGCTGCCGCCAG	Rv primer for TS7 containing NheI site
JVO-21361	TCCAGTGAAAAGTTCTTCTCCTTTGCTAGCCATCTTTTGCGCTGCCGCCAG	Rv primer for fusion PCR of TS7 with 30 nt overlap GFP (pXG10)
JVO-21366	CGTCTTTGAATGTCTGTAAACAGAGGAGATACAG	Sense oligo to generate TS7-Mut_9
JVO-21367	CTCTGTTTACAGACATTCAAAGACGACAATAAGTG	Antisense oligo to generate TS7-Mut_9
JVO-21368	CGTCTTTGTATGCCTGTAAACAGAGGAGATAC	Sense oligo to generate TS7-Mut_13
JVO-21369	CCTCTGTTTACAGGCATACAAAGACGACAATAAG	Antisense oligo to generate TS7-Mut_13
JVO-21370	GTATGTCTGTCAACAGAGGAGATACAGAATGAC	Sense oligo to generate TS7-Mut_18
JVO-21371	CTGTATCTCCTCTGTTGACAGACATACAAAGAC	Antisense oligo to generate TS7-Mut_18
JVO-21511	GTGAGCAAAGGTGAAGAAGATAATATG	Fw oligo for amplification of mNeon
JVO-21512	CGCCATATTATCTTCTTCACCTTTGCTCACCATCTTTTGCGCTGCCGCCAG	Rv oligo for fusion PCR of TS7 with 30 nt overlap mNeonGreen
PZE-CAT	TGGGATATATCAACGGTGGT	For sequencing of pXG10-derived constructs

Sequences are given in 5’ to 3’ direction; Fw indicates forward, Rv indicates reverse.

Table S4. List of plasmids used in this study

Plasmid Number	Insert name	Backbone/ Marker	Oligos for insert amplification	Cloning sites	Description
pPS002	TS7	pXG10-SF/Cm	JVO-21317/ JVO-21318	NsiI/NheI	TS7 insert was cloned from pNP1-TS7 obtained from Addgene (Plasmid #107355)
pPS007	TS7-GFP	pNP1/Amp			Addgene Plasmid #107355
pPS013	TS7-Mut_18	pXG10-SF/Cm	JVO-21370/ JVO-21371		Mutations induced into pXG10-TS7 (pPS2) at pos 18 of stem
pPS014	TS7	pXG10/Cm	JVO-21317/ JVO-20632/ JVO-21511/ JVO-21512/	NheI/XbaI	Template TS7: pPS002 Fused to mNeon (from pKAS32mNeon) followed by cloning into in pXG10-SF
pPS016	TS7-Mut_9	pXG10-SF/Cm	JVO-21366/ JVO-21367		Single site mutation induced into TS7
pPS017	TS7-Mut_13	pXG10-SF/Cm	JVO-21368/ JVO-21369		Single site mutation induced into TS7
pPS020	TS7-mCherry	pXG10-mCherry/Cm	JVO-19329/ JVO-19330	NheI/ XbaI	Template pSDM007 Backbone pPS002
pPS035	TS7-Mut_9	pXG10-mNeon/Cm	JVO-21366/ JVO-21367		Template pPS014
pPS036	TS7-Mut_9	pXG10-mCherry/Cm	JVO-21366/ JVO-21367		Template pPS020

Template indicates template plasmid used for insert synthesis. Backbone indicates vector.

Table S5. List of strains used in this study

Name	Strain	Plasmid	Marker	Description
**JVS-1574**	*S. typhimurium* SL1344			*Wild-type* strain
**JVS-3013**	*S.typhimurium* SL1344	pKD46	Str/Amp	JVS-1574 transformed with pKD46
**JVS-11959**	*S typhimurium* SL1344 Δ*sbmA*		Km	JVS-3013 was electroporated with a PCR product of JVO-16358/16359 template pKD4 Verification JVO-16360/16361
**PSS-024**	*S. typhimurium* SL1344	pPS002	Cm	JVS-1574 was transformed with pPS002
**PSS-032**	*S. typhimurium* SL1344	pPS013	Cm	JVS-1574 was transformed with pPS013
**PSS-037**	*S. typhimurium* SL1344	pPS016	Cm	JVS-1574 was transformed with pPS0016
**PSS-038**	*S. typhimurium* SL1344	pPS017	Cm	JVS-1574 was transformed with pPS017
**PSS-077**	*S. typhimurium* SL1344 Δ*sbmA*	pPS016	Cm	JVS-11959 was transformed with pPS016
**PSS-088**	*S. typhimurium* SL1344	pPS036	Cm	JVS1574 was transformed with pPS036
**PSS-089**	*S. typhimurium* SL1344	pPS035	Cm	JVS1574 was transformed with pPS035
**Top10**	*E. coli*			Purchased from Invitrogen
**JVS-12547**	*E. coli* K12 BW25113			*Wild-type* strain
**PSS-049**	*E. coli* BW25113	pPS016	Cm	JVS-12547 was transformed with pPS016
**JVS-5709**	*E. coli* K12 MG1655			*Wild-type* strain
**PSS-017**	*E. coli* MG1655	pPS002	Cm	JVS-5709 was transformed with pPS002
**PSS-063**	*E. coli* MG1655	pPS016	Cm	JVS5709 was transformed with pPS016
**JVS-12054**	*E. coli* UPEC 536			*Wild-type* strain
**PSS-047**	*E. coli* UPEC 536	pPS016	Cm	JVS-12054 was transformed with pPS016

Cm indicates chloramphenicol; Amp, ampicillin; Km, kanamycin; Str, streptomycin.

Table S6. List of CPP-PNAs used in this study

Internal Number	Name	PNA Sequence (N to C terminus)
JVpna-14	PNA_ctrl	TCACTATCTC
JVpna-872	PNA_toe	CAAAGACGACA
JVpna-819	KFF-PNA_mid	ACAGACATACA
**Internal Number**	**Name**	**CPP Sequence (N to C terminus)**
**Set 1:** CPPs were conjugated to PNA_toe sequence CAAAGACGACA		
JVpna-818	KFF-PNA_toe	KFFKFFKFFK (KFF)
JVpna-855	RXR-PNA_toe	RXRRXRRXRRXRXB (RXR)
JVpna-885	ANT- PNA_toe	RQIKIWFQNRRMKWKK (ANT)
JVpna-886	TAT- PNA_toe	GRKKRRQRRRYK (TAT)
JVpna-887	Seq118- PNA_toe	RRRQRRKKRGY (Seq118)
JVpna-888	Seq35- PNA_toe	RKKRRQRR (Seq35)
JVpna-889	Seq229- PNA_toe	GRRRRRRRRRPPQ (R9-TAT)
JVpna-890	R5- PNA_toe	RRRRR (R5)
JVpna-891	L5a-PNA_toe	RRWQW (L5a)
JVpna-892	Seq23-PNA_toe	VALLPAVLLA (Seq23)
**Set 2:** CPPs were conjugated to *acpP* targeting PNA sequence CTCATACTCT		
JVpna-18	RXR-*acpP*	RXRRXRRXRRXRXB (RXR)
JVpna-21	KFF-*acpP*	KFFKFFKFFK (KFF)
JVpna-115	Seq118- *acpP*	RRRQRRKKRGY (Seq118)
JVpna-119	Seq23- *acpP*	VALLPAVLLA (Seq23)
JVpna-120	R9TAT-*acpP*	GRRRRRRRRRPPQ (R9-TAT)
JVpna-143	TAT-*acpP*	GRKKRRQRRRYK (TAT)
JVpna-1031	ANT-*acpP*	RQIKIWFQNRRMKWKK (ANT)
JVpna-1032	R5-*acpP*	RRRRR (R5)
JVpna-1033	Seq35-*acpP*	RKKRRQRR (Seq35)
JVpna-1034	L5a-*acpP*	RRWQW (L5a)
**Set 3:** CPPs were conjugated to non-targeting PNA sequence TCACTATCTC		
JVpna-2	ANT-PNA_ctrl	RQIKIWFQNRRMKWKK (ANT)
JVpna-19	RXR- PNA_ctrl	RXRRXRRXRRXRXB (RXR)
JVpna-22	KFF- PNA_ctrl	KFFKFFKFFK (KFF)
JVpna-97	TAT- PNA_ctrl	GRKKRRQRRRYK (TAT)
JVpna-183	seq118- PNA_ctrl	RRRQRRKKRGY (Seq118)
JVpna-184	seq35- PNA_ctrl	RKKRRQRR (Seq35)
JVpna-186	R9TAT- PNA_ctrl	GRRRRRRRRRPPQ (R9-TAT)
JVpna-1157	L5a- PNA_ctrl	RRWQW (L5a)
JVpna-1325	R5- PNA_ctrl	RRRRR (R5)

X indicates 6-amino-hexanoic acid, B is ß-alanine
